# Supplementary material for: Addressing Trauma and Building Resilience in Children and Families: Standardized Patient Cases for Pediatric Residents
Source: MedEdPORTAL. 2021 Nov 8;17:11193. doi: 10.15766/mep_2374-8265.11193 (PMC8592119; doi:10.15766/mep_2374-8265.11193)
Supplement: Supplementary file 1 — Case 1.docxCase 2.docxCase 3.docxResource Packet.docxOrientation Slides.pptxWays to Ask About Trauma.mp4NCTSN Encounter Learner Handout.docxDe-escalation Strategies.mp4Scenario 1 Evaluation Checklist.docxScenario 2 Evaluation Checklist.docxScenario 3 Evaluation Checklist.docxDebrief Instructions.docxPresurvey.docxPostsurvey.docxEncounter-Specific Survey.docx [file mep_2374-8265.11193-s001.zip › _Educational_Summary_Report_11193.pdf]

MedEdPORTAL® | The AAMC Journal of Teaching and Learning Resources

Original Publication

Open Access

# Addressing Trauma and Building Resilience in Children and Families: Standardized Patient Cases for Pediatric Residents

M. Cooper Lloyd, MD, MPH*, Jessica Ratner, MD, Jaime La Charite, MD, MPH, Robin Ortiz, MD, Sean Tackett, MD, MPH, Leonard Feldman, MD, Barry S. Solomon, MD, MPH, Nicole Shilkofski, MD, MEd

*Corresponding author: m.cooper.lloyd@vumc.org

## Abstract

**Introduction:** Adverse childhood experiences (ACEs) and trauma are common and can negatively impact children's health. Standardized patient (SP) learning may provide trainees with knowledge and skills to screen for and manage ACEs, apply trauma-informed care approaches, and teach resilience strategies. **Methods:** With content experts, we developed three SP cases based on common clinical encounters, as well as didactic and debriefing materials. Case 1 focused on somatic symptoms in an adolescent with ACEs, case 2 focused on an ACE disclosure by a parent, and case 3 focused on de-escalation. The workshop required facilitators, SPs, simulation exam room and meeting space, and audiovisual equipment. It lasted 4 hours and included an orientation (1 hour), the three SP cases (totaling 2 hours), and group debriefing (1 hour). **Results:** We conducted five identical workshops with 22 pediatric residents. Participants responded favorably to case fidelity and applicability to their clinical work. Resident mean self-assessment scores improved significantly from baseline. Specifically, we assessed comfort with inquiring about and discussing ACEs, explaining the health impacts of trauma, identifying protective factors, resilience counseling, and de-escalation. Over 90% of responses indicated that residents were likely to apply what they had learned to their clinical practice. **Discussion:** These findings demonstrate that our SP cases were well received and suggest that such curricula can help pediatric residents feel more prepared to address trauma and promote resilience. Future work will assess these outcomes, as well as behavior change, in a larger sample to further substantiate these promising findings.

### Keywords
Adverse Childhood Experiences, Trauma-Informed Care, Resilience, Standardized Patient, De-escalation, Communication Skills, Pediatrics, Simulation

## Educational Objectives

By the end of this activity, learners will be able to:

1. Ask patients and families about adverse childhood experiences (ACEs) and trauma.
2. Discuss ACEs and trauma with patients and families.
3. Explain the impact of trauma on physical and emotional health.
4. Identify protective factors that build resilience.
5. Counsel patients and families about building resilience.
6. Use trauma-informed principles to de-escalate an escalated patient.

**Citation:**
Lloyd MC, Ratner J, La Charite J, et al. Addressing trauma and building resilience in children and families: standardized patient cases for pediatric residents. *MedEdPORTAL*. 2021;17:11193.
https://doi.org/10.15766/mep_2374-8265.11193

## Introduction

Trauma in childhood is associated with negative physical and mental health outcomes across the life span.[1,2] Adverse childhood experiences (ACEs)—10 types of adversity originally defined by Felitti and colleagues—are common, with sources suggesting that about two-thirds of the U.S. population has experienced at least one ACE.[1,2] In recent years, health care systems have increasingly recognized the importance of trauma-informed care (TIC), an approach to service delivery that infuses awareness of trauma into clinical interactions and attempts to assist in recovery while avoiding retraumatization.[3] Resilience—the ability to adapt to adversity—is a buffer against ACEs that can be fostered, particularly through nurturing relationships and active coping strategies.[4-6] Positive supports and resiliency have been shown to decrease the likelihood of mental health and

Copyright © 2021 Lloyd et al. This is an open-access publication distributed under the terms of the Creative Commons Attribution license.

substance use disorders, as well as improve health and school engagement.[7-9]

Despite the extensive impacts of ACEs on health outcomes and the potential to support recovery, pediatric trainees receive little training on ACEs or resilience. In one study, 75% of general pediatricians were unfamiliar with the original ACEs data, and one-third reported not asking about ACEs in clinical practice.[10,11] Similarly, in a survey of family medicine residents, 2% reported screening for ACEs, and 65% did not feel confident in screening. Eighty-five percent of residents, however, felt it was their role to screen.[11] A survey at Johns Hopkins Hospital in Baltimore, Maryland, and Johns Hopkins All Children's Hospital in St. Petersburg, Florida, showed that 87% of pediatric residents in these programs desired additional training on ACEs and resilience (unpublished data, August 2018).

Though several curricula have been designed to address ACEs and TIC, most have been limited in format and trainee demographic. Educators have developed brief didactic sessions on ACEs,[12-14] case-based discussions,[15,16] games,[17] practice sessions or role-plays,[18] and simulation.[19] These curricula have generally been well received and have shown promising outcomes.[17] However, most programs have been conducted with medical students or nonpediatric residents and have focused on only one element of ACEs and TIC, such as screening or exam skills. Consequently, trainees request additional training and practice in clinical settings.[15] The one standardized patient (SP) intervention developed for pediatric residents taught de-escalation techniques but did not embed these in the context of TIC.[20]

Given the lack of comprehensive programs for pediatric residents on these topics, we designed a novel, longitudinal, multimodal curriculum for pediatric residents with the goal of strengthening knowledge, confidence, and practical skills related to trauma and resilience. As part of this curriculum, we developed three SP cases embedded in a half-day training workshop for pediatric residents to practice skills and build confidence with trauma screening, resiliency counseling, and de-escalation techniques. SP-based learning is an effective method of improving resident performance and confidence in managing difficult patient encounters.[21] Furthermore, this format draws on Kolb's experiential learning theory, which involves experiencing something new, reflection on the experience, conceptualization, and application to real-world experiences.[22] To our knowledge, our educational material is the first designed to teach pediatric residents about ACEs, TIC, and resiliency using SPs.

## Methods

### Case Development

We chose topics for SP cases that reflected a variety of trauma-related clinical scenarios frequently encountered by pediatric trainees. In particular, we focused on developing skills that residents at our institution requested in a needs assessment distributed during the curriculum development process.

Cases included the following:

- Case 1: Somatic symptoms in an adolescent with ACEs (Appendix A).
- Case 2: ACE disclosure by a parent (Appendix B).
- Case 3: De-escalation (Appendix C).

While they were residents, authors M. Cooper Lloyd, Jessica Ratner, Jaime La Charite, and Robin Ortiz drafted SP cases and supporting material based on clinical experiences and training in TIC. Materials were reviewed by pediatrics and child psychiatry faculty with expertise in TIC, simulation including SP cases, and curriculum development and were modified to ensure appropriate content. Simulation center staff and SPs also reviewed scenarios to ensure feasibility. During the first workshops, SP encounters were video-recorded and reviewed to ensure consistency and accuracy; in addition, residents and faculty observed all encounters in real time to allow for immediate feedback to SPs. Observations from residents and faculty and feedback from participants and SPs informed minor modifications to the scenarios and workshop materials.

### Equipment/Environment

Simulated clinical exam rooms and conference room space were needed to run the workshop. Audiovisual setup with a computer and audio was also needed for the PowerPoint presentation and videos used in the orientation.

### Personnel

At least one facilitator was required to lead didactic instruction and debriefing. Facilitators should have basic knowledge of ACEs, TIC, and resilience and should review the cases and materials prior to facilitating. In addition, they should have basic skills in facilitating group discussion in order to lead debriefing. Our sessions were facilitated by at least one resident team member, assisted by a faculty member. Resident team members had content expertise as a result of participation in the American Academy of Pediatrics' Trauma-Informed Pediatric Provider Course in 2018.[23] One faculty member had expertise in simulation and resident education. A second faculty member had content expertise in pediatric TIC.

Copyright © 2021 Lloyd et al. This is an open-access publication distributed under the terms of the Creative Commons Attribution license. 

Additional simulation personnel were helpful to proctor and assist with the flow of learners through the cases. We used a ratio of one proctor per six learners. The facilitator could serve as a proctor if they were also trained in facilitating SP encounters.

Each case required one SP, as indicated below:

- Case 1 (Mariana): preferably a young adult female actor but could be altered to accommodate a male actor.
- Case 2 (Mr./Mrs. Carver): an adult female or male actor.
- Case 3 (Jordan): a young adult female or male actor.

Simulation staff selected SPs using the above criteria. Prior to implementation, the curriculum team and simulation staff held a 3-hour training for actors. Actors were sent case materials to read before the training. During the training, resident team members modeled cases, and actors asked clarifying questions about content and desired patient portrayal. Our curriculum team also reviewed evaluation checklists (more details below in Assessment) with the SPs and answered questions about providing feedback to residents. For institutions not able to hire SPs, faculty members or trainees could play these roles, using training methods similar to those described above.

### Workshop Implementation

Junior and senior pediatric residents participated in a half-day (4-hour) workshop that included an orientation, the three SP cases, and a group debriefing. Residents did not need prerequisite knowledge but were asked to read a packet of materials, including short articles on trauma and resilience (approximately 20 minutes of reading) as well as a list of optional patient resources (requiring about 20 minutes to explore), prior to the session (Appendix D). We also collected several optional handouts on age-appropriate coping strategies, including mindful breathing, naming emotions, and identifying personal strengths, from organizations such as Sesame Street in Communities and Anxiety Canada, for residents to review prior to the session.

*Orientation and didactic training:* We conducted a 1-hour orientation and training at the beginning of the session to review key knowledge and skills to prepare residents for the cases. A PowerPoint presentation (Appendix E) guided the residents through the following activities:

- Reviewing common trauma-related symptoms.
- Watching a video that modeled ways to ask about trauma (Appendix F), followed by group discussion.
- Listening to an audio recording of a model encounter from the National Child Traumatic Stress Network (NCTSN) Learning Center (optional activity).[24] A handout for learner note-taking was used to facilitate engagement (Appendix G). This clip was divided into two sections. During the first section, residents noted trauma symptoms exhibited by the patient and ways that the pediatrician asked about trauma exposure. During the second section, residents noted steps that the pediatrician took after the disclosure of trauma. Each clip was followed by group discussion.
- Reviewing evidence-based strategies to promote resilience and recovery.
- Watching a video that portrayed de-escalation techniques (Appendix H), followed by group discussion.

Videos in Appendices F and H were created by authors M. Cooper Lloyd, Jessica Ratner, Jaime La Charite, and Robin Ortiz and can also be found via hyperlinks in the presentation.

*SP encounters:* Following orientation and training, residents rotated individually through each of the three SP cases. A total of 36 minutes was appropriate for each case (20 minutes for completion of scenario, 8 minutes for SPs to complete evaluation checklists, and 8 minutes for feedback by the SP). Prior to beginning each case, residents received instructions (Appendices A-C) that oriented them to the scenario and provided objectives. Residents were permitted to use the resources packet (optional material in Appendix D) during encounters, if desired; future facilitators should feel free to find their own content to supplement listed resources. After completing the encounter, residents left the room, and SPs used evaluation checklists (Appendices I-K; see Assessment, below, for details) to guide their assessment. Residents then returned to the room to receive direct, formative feedback from SPs.

*Debriefing:* After completion of all cases, residents reconvened for a 1-hour group debriefing session. The facilitator guided discussion using debriefing questions focused on residents' experiences practicing new communication strategies, prior relevant clinical experiences, and self-care strategies while caring for trauma-affected patients (Appendix L). During the debrief, we also reviewed patient handouts with relevant community resources.

### Assessment

We developed formative evaluation checklists (Appendices I-K) for all three cases to guide SPs' assessment of resident performance. Checklists included skills, based on scenario objectives and measured with 5-point Likert scales, that our curriculum team deemed critical to successful navigation of each case. During pilot sessions, SPs were given the opportunity to

Copyright © 2021 Lloyd et al. This is an open-access publication distributed under the terms of the Creative Commons Attribution license.

ask questions about evaluation checklists and expressed comfort with the format used for assessment.

In addition, residents completed presurveys (Appendix M) and postsurveys (Appendix N) to assess the effectiveness of the workshop in achieving objectives. Surveys included questions, measured with 5-point Likert scales, about how often participants considered ACEs when evaluating a chief complaint, as well as comfort doing each of the following with patients: discussing ACEs, asking about experience of trauma, explaining trauma's impact on health, identifying protective factors/counseling on resilience, and de-escalation. Following participation in each case, residents also completed encounter-specific surveys (Appendix O), which included questions about learner comfort, case realism, and applicability to practice.

We developed the surveys based on learning objectives and for iterative feedback purposes. Content experts reviewed the surveys for appropriate content. We distributed the surveys via email using Qualtrics. Each participant was assigned a confidential ID number that allowed linkage of surveys and protected anonymity. The Johns Hopkins University School of Medicine Institutional Review Board reviewed the study proposal and considered data collection to be exempt, given that it was completed as part of a quality improvement project (IRB00177294, 2018).

Stata 13 (Release 13, StataCorp) was used for data analysis. We conducted descriptive analysis on all survey response items. To compare the pre- and postworkshop survey median scores of participants who completed both surveys ($n = 14$), we used the paired Student $t$ test. A sensitivity analysis using an unpaired $t$ test of pre/post data from all participants ($n = 22$) was also conducted.

## Results

Curricular Implementation

We conducted identical in-person workshops five times from March 2019 to March 2020, with groups of four to five participants in each session for a total of 22 residents. Participants were PGY 2-4 residents in categorical and combined pediatrics residency programs at Johns Hopkins Hospital. We asked residents to participate while on rotations in adolescent medicine, mental health, or behavior and development due to these rotations' relevance to curricular material.

Curricular Program Evaluation

Data collected in encounter-specific surveys show acceptable realism and applicability of each of the SP cases (Table 1). Nineteen participants completed encounter-specific surveys, with the exception of case 1 ($n = 18$). Mean scores were between 4 and 5 (with 5 being most positive) across all three cases for questions regarding realism/fidelity, applicability to participants' clinical work, and likelihood of applying learning to participants' work. Results were consistent across cases. Across all scenarios, 93% of responses (52 out of 56) indicated that residents were likely or very likely to apply what they had learned to their work.

Among 22 resident participants, 21 completed preworkshop surveys, and 19 completed postworkshop surveys, of which 14 paired observations were available for analysis. Four participants from the first workshop were not assigned IDs, so these survey results could not be paired. Table 2 summarizes paired, participant-reported comfort using various trauma-informed skills before and after participation in the workshop. Comfort using all skills increased significantly after participation, with the greatest increases seen in identifying protective factors, counseling regarding resilience, consideration of ACEs and/or trauma when evaluating a patient's chief complaint, and de-escalating an upset patient or parent. Analyzing unpaired means for pre/post data from all 22 participants yielded similar results.

## Discussion

A growing body of literature has identified the importance for health care professionals of understanding ACEs, TIC, and resilience and effectively communicating with patients about these topics. These competencies are particularly important in pediatrics, where clinicians routinely encounter children who may be particularly vulnerable to toxic stress and uniquely able to benefit from early intervention. Related educational programs for

**Table 1.** Mean Scores Reflecting Realism and Applicability of Standardized Encounters

| | Case 1 ($n = 18$) | | Case 2 ($n = 19$) | | Case 3 ($n = 19$) | |
|---|---|---|---|---|---|---|
| **Survey Question** | ***M*** | ***SD*** | ***M*** | ***SD*** | ***M*** | ***SD*** |
| To what extent do you think that this exercise simulated a real clinical encounter? (1 = *not at all,* 5 = *a lot*) | 4.33 | 0.75 | 4.37 | 0.74 | 4.16 | 1.14 |
| How applicable was this exercise to your work? (1 = *not applicable at all,* 5 = *very applicable*) | 4.50 | 0.96 | 4.63 | 0.74 | 4.21 | 0.83 |
| How likely are you to apply what you learned in this exercise to your work? (1 = *very unlikely,* 5 = *very likely*) | 4.22 | 0.79 | 4.47 | 0.75 | 4.37 | 0.74 |

Copyright © 2021 Lloyd et al. This is an open-access publication distributed under the terms of the Creative Commons Attribution license. 

**Table 2.** Mean Difference in Resident Scores Pre/Post Encounters ($N = 14$)

| Survey Question | Presurvey *M* (95% CI) | Postsurvey *M* (95% CI) | Mean Difference | *p* |
|---|---|---|---|---|
| 1. How often do you consider ACEs and/or trauma when evaluating a patient's chief complaint? | 3.1 (2.5-3.7) | 4.1 (3.6-4.5) | 1.0 | .004[a] |
| 2. How comfortable do you feel discussing ACEs and/or trauma with your patients/parents? | 3.5 (3.0-4.0) | 4.1 (3.7-4.4) | 0.6 | .006[a] |
| 3. How comfortable do you feel asking your patients if they have experienced an ACE and/or traumatic event? | 3.6 (3.3-4.0) | 4.0 (3.7-4.3) | 0.4 | .019[a] |
| 4. How comfortable do you feel explaining to patients/parents how traumatic experiences impact health? | 3.4 (2.8-3.9) | 4.3 (3.9-4.6) | 0.9 | .001[a] |
| 5. How comfortable do you feel identifying protective factors and counseling patients/parents on how to foster resilience? | 2.5 (2.0-3.0) | 3.8 (3.4-4.2) | 1.3 | <.001[a] |
| 6. How comfortable do you feel de-escalating an escalated patient or parent? | 3.0 (2.4-3.6) | 4.0 (3.6-4.4) | 1.0 | <.001[a] |

Abbreviation: ACE, adverse childhood experience.
[a]$p < .05$.

pediatric residents have not yet included SP exercises to practice relevant skills, though such exercises are increasingly identified as a need.[25] This gap in training motivated us to create three scenarios designed to increase resident comfort and confidence in addressing these topics in clinical settings.

Our evaluation demonstrates that residents found the SP scenarios highly realistic, representative of clinical situations they encounter, and applicable to their work. Furthermore, our evaluation demonstrates significant changes in resident self-reported comfort asking about and discussing ACEs and trauma, explaining the impacts of trauma on health, identifying protective factors, counseling about resilience, and de-escalating an escalated patient. An increase in self-reported comfort with these skills suggests an increase in self-efficacy. A prior meta-analysis of SP curricula indicated that working with SPs improves self-efficacy as well as clinical competence among health professions students.[26] Further evaluation of our curriculum is needed to determine whether the desired skills are attained in the simulation environment and whether they translate to clinical practice.

The cases have proven versatile. We have developed telemedicine versions of the cases in the setting of COVID restrictions; a pilot study of these versions is ongoing. The cases could also be adapted as role-plays in order to accommodate resource or setting constraints. If adapted as role-plays, further evaluation would be needed to determine whether the cases remain effective teaching tools when used more informally, without trained SPs.

Several lessons have emerged from development and implementation of these scenarios. Case 3, focused on de-escalation, has required the greatest iteration. We used a quality improvement approach across multiple sessions to guide improvement. In earlier sessions, we found that SPs were not acting escalated in the ways we had envisioned, and residents confirmed that performances were variable across actors. For example, some SPs portrayed sullenness without heightened or dysregulated emotions. Based on this feedback, we edited SP training materials to outline specific ways that actors could show escalation (e.g., raised voice, kicking the wall, pounding fist on table, etc.). We also met with SPs before subsequent sessions to clarify expectations for how to portray escalation. Our direct observations as well as resident qualitative feedback revealed improvement. Other programs may benefit from upfront discussions regarding acting expectations in case 3, including specific actions that demonstrate emotional dysregulation.

Logistics provided a second challenge. The workshop requires approximately 4 hours of protected time for residents. Identifying multiple half-days during which residents could be released from clinical activities for participation proved challenging. While we selected three rotations from which residents could be released periodically during the academic year, other programs may consider releasing an entire residency class from clinical duties for a half-day session to maximize participation while minimizing costs associated with running multiple small sessions.

Finances challenged us as well. Running SP sessions in a simulation center with trained actors requires ongoing capital. While our sessions were grant funded, other programs may need to search creatively for funding. Alternatively, programs might consider adapting the scenarios as role-plays as described above, with learners serving as both patient actors and clinicians.

There are limitations to our curriculum and evaluation approach. First, case 1 focuses on an adolescent Latina to address the trauma associated with immigration and the ACE of parental separation. This scenario may not be generalizable to every community, but it can be easily modified for other immigrant communities. Second, our evaluation was limited by our small number of participants and lack of a high response rate. Third, while the intervention yielded statistically significant changes

Copyright © 2021 Lloyd et al. This is an open-access publication distributed under the terms of the Creative Commons Attribution license.

in self-reported comfort, the lack of a control group limited our ability to make comparisons with those who did not receive the intervention. Fourth, we only assessed change in participants' subjective comfort with relevant skills rather than real-world behavior change. Despite these limitations, our findings suggest that the curricular resources met our intended goals.

Future directions include assessment of role-play and telemedicine versions of the cases to allow for greater generalizability and sustainability. Furthermore, as these SP encounters are part of a comprehensive, longitudinal curriculum, we plan to assess reported behavior changes as a result of SP case participation in future analyses. Positive results will be used to advocate for sustainable funding and permanently embedding these learning opportunities in resident curricula.

This learning exercise joins a small but growing literature within medical education designed to equip pediatric residents with the skills needed to address trauma and help families build resilience. These exercises can be adapted by other pediatric residency programs to enhance essential communication skills about these subjects. With intentional training and practice, we can better prepare a new generation of trauma-informed pediatric providers to meet the needs of children and families who have experienced trauma.

## Appendices

A. Case 1.docx
B. Case 2.docx
C. Case 3.docx
D. Resource Packet.docx
E. Orientation Slides.pptx
F. Ways to Ask About Trauma.mp4
G. NCTSN Encounter Learner Handout.docx
H. De-escalation Strategies.mp4
I. Scenario 1 Evaluation Checklist.docx
J. Scenario 2 Evaluation Checklist.docx
K. Scenario 3 Evaluation Checklist.docx
L. Debrief Instructions.docx
M. Presurvey.docx
N. Postsurvey.docx
O. Encounter-Specific Survey.docx

*All appendices are peer reviewed as integral parts of the Original Publication.*

**M. Cooper Lloyd, MD, MPH:** Resident, Departments of Internal Medicine and Pediatrics, Johns Hopkins University School of Medicine; ORCID: https://orcid.org/0000-0001-7364-6716

**Jessica Ratner, MD:** Resident, Departments of Internal Medicine and Pediatrics, Johns Hopkins University School of Medicine

**Jaime La Charite, MD, MPH:** Resident, Departments of Internal Medicine and Pediatrics, Johns Hopkins University School of Medicine

**Robin Ortiz, MD:** Resident, Departments of Internal Medicine and Pediatrics, Johns Hopkins University School of Medicine; ORCID: https://orcid.org/0000-0002-0957-5850

**Sean Tackett, MD, MPH:** Associate Professor, Department of Medicine, Johns Hopkins Bayview Medical Center and Johns Hopkins University School of Medicine; Core Faculty, Biostatistics, Epidemiology, and Data Management Core, Johns Hopkins University School of Medicine; ORCID: https://orcid.org/0000-0001-5369-7225

**Leonard Feldman, MD:** Associate Professor, Departments of Internal Medicine and Pediatrics, Johns Hopkins University School of Medicine; ORCID: https://orcid.org/0000-0002-4444-6624

**Barry S. Solomon, MD, MPH:** Professor, Department of Pediatrics, Johns Hopkins University School of Medicine

**Nicole Shilkofski, MD, MEd:** Associate Professor, Department of Pediatrics, Johns Hopkins University School of Medicine; ORCID: https://orcid.org/0000-0003-0000-0871

### Acknowledgments

Drs. Lloyd, Ratner, and La Charite contributed equally to this work.

The authors would like to acknowledge Rachel Cane, MD, PhD, for her assistance with workshop facilitation, Tiffany Mark, MD, for her help developing evaluation checklists, and Heather Forkey, MD, for reviewing the simulation cases.

### Disclosures

None to report.

### Funding/Support

Dr. Leonard Feldman received funding from the Health Resources and Services Administration (Grant #D58HP37574). Dr. Barry S. Solomon received funding from the Johns Hopkins Medicine Primary Care Consortium. Dr. Nicole Shilkofski received funding from the Association for Pediatric Program Directors.

### Ethical Approval

The Johns Hopkins University School of Medicine Institutional Review Board deemed further review of this project not necessary.

## References

1. Oral R, Ramirez M, Coohey C, et al. Adverse childhood experiences and trauma informed care: the future of health care. *Pediatr Res*. 2016;79(1):227-233. https://doi.org/10.1038/pr.2015.197

Copyright © 2021 Lloyd et al. This is an open-access publication distributed under the terms of the Creative Commons Attribution license.

2. Felitti VJ, Anda RF, Nordenberg D, et al. Relationship of childhood abuse and household dysfunction to many of the leading causes of death in adults: the Adverse Childhood Experiences (ACE) Study. *Am J Prev Med*. 1998;14(4):245-258. https://doi.org/10.1016/S0749-3797(98)00017-8
3. Anda RF, Butchart A, Felitti VJ, Brown DW. Building a framework for global surveillance of the public health implications of adverse childhood experiences. *Am J Prev Med*. 2010;39(1):93-98. https://doi.org/10.1016/j.amepre.2010.03.015
4. Chandler GE, Roberts SJ, Chiodo L. Resilience intervention for young adults with adverse childhood experiences. *J Am Psychiatr Nurses Assoc*. 2015;21(6):406-416. https://doi.org/10.1177/1078390315620609
5. Traub F, Boynton-Jarrett R. Modifiable resilience factors to childhood adversity for clinical pediatric practice. *Pediatrics*. 2017;139(5):e20162569. https://doi.org/10.1542/peds.2016-2569
6. Leitch L. Action steps using ACEs and trauma-informed care: a resilience model. *Health Justice*. 2017;5:5. https://doi.org/10.1186/s40352-017-0050-5
7. Wrenn GL, Wingo AP, Moore R, et al. The effect of resilience on posttraumatic stress disorder in trauma-exposed inner-city primary care patients. *J Natl Med Assoc*. 2011;103(7):560-566. https://doi.org/10.1016/S0027-9684(15)30381-3
8. Earls MF. Trauma-informed primary care: prevention, recognition, and promoting resilience. *N C Med J*. 2018;79(2):108-112. https://doi.org/10.18043/ncm.79.2.108
9. Sege R, Bethell C, Linkenbach J, Jones J, Klika B, Pecora PJ. *Balancing Adverse Childhood Experiences With HOPE: New Insights Into the Role of Positive Experience on Child and Family Development*. The Medical Foundation; 2017.
10. Kerker BD, Storfer-Isser A, Szilagyi M, et al. Do pediatricians ask about adverse childhood experiences in pediatric primary care? *Acad Pediatr*. 2016;16(2):154-160. https://doi.org/10.1016/j.acap.2015.08.002
11. Tink W, Tink JC, Turin TC, Kelly M. Adverse childhood experiences: survey of resident practice, knowledge, and attitude. *Fam Med*. 2017;49(1):7-13.
12. Schiff DM, Zuckerman B, Hutton E, Genatossio C, Michelson C, Bair-Merritt M. Development and pilot implementation of a trauma-informed care curriculum for pediatric residents. *Acad Pediatr*. 2017;17(7):794-796. https://doi.org/10.1016/j.acap.2017.03.011
13. Schmitz A, Light S, Barry C, Hodges K. Adverse childhood experiences and trauma-informed care: an online module for pediatricians. *MedEdPORTAL*. 2019;15:10851. https://doi.org/10.15766/mep_2374-8265.10851
14. Shamaskin-Garroway AM, McLaughlin EA, Quinn N, Buono FD. Trauma-informed primary care for medical residents. *Clin Teach*. 2020;17(2):200-204. https://doi.org/10.1111/tct.13082
15. Pletcher BA, O'Connor M, Swift-Taylor ME, DallaPiazza M. Adverse childhood experiences: a case-based workshop introducing medical students to trauma-informed care. *MedEdPORTAL*. 2019;15:10803. https://doi.org/10.15766/mep_2374-8265.10803
16. Sonney J, Willgerodt M, Lindhorst T, Brock D. Elizabeth: typical or troubled teen? A training case for health professionals to recognize and report child maltreatment. *MedEdPORTAL*. 2018;14:10712. https://doi.org/10.15766/mep_2374-8265.10712
17. Stefanski K, Mason K. Acing education: pilot curriculum on adverse childhood experiences. *Med Educ*. 2017;51(11):1167-1168. https://doi.org/10.1111/medu.13436
18. Elisseou S, Puranam S, Nandi M. A novel, trauma-informed physical examination curriculum for first-year medical students. *MedEdPORTAL*. 2019;15:10799. https://doi.org/10.15766/mep_2374-8265.10799
19. Wen FK, Miller-Cribbs JE, Coon KA, Jelley MJ, Foulks-Rodriguez KA. A simulation and video-based training program to address adverse childhood experiences. *Int J Psychiatry Med*. 2017;52(3):255-264. https://doi.org/10.1177/0091217417730289
20. Hilgenberg SL, Bogetz AL, Leibold C, Gaba D, Blankenburg RL. De-escalating angry caregivers: a randomized controlled trial of a novel communication curriculum for pediatric residents. *Acad Pediatr*. 2019;19(3):283-290. https://doi.org/10.1016/j.acap.2018.10.005
21. Wells J, Cronk NJ. Practice makes perfect: training residents in difficult encounters. *PRiMER*. 2020;4:2. https://doi.org/10.22454/PRiMER.2020.924066
22. Kolb DA. *Experiential Learning: Experience as the Source of Learning and Development*. Prentice-Hall; 1983.
23. American Academy of Pediatrics. Trauma-informed pediatric provider. Course offered: April 2018; Houston, TX.
24. Models of trauma-informed integrated care part II: identifying and responding to early childhood trauma in the pediatric setting. National Child Traumatic Stress Network Learning Center. Accessed July 15, 2018. https://learn.nctsn.org/mod/nctsnwebinar/view.php?id=9542
25. Chokshi B, Walsh K, Dooley D, Falusi O, Deyton L, Beers L. Teaching trauma-informed care: a symposium for medical students. *MedEdPORTAL*. 2020;16:11061. https://doi.org/10.15766/mep_2374-8265.11061
26. Oh PJ, Jeon KD, Koh MS. The effects of simulation-based learning using standardized patients in nursing students: a meta-analysis. *Nurse Educ Today*. 2015;35(5):e6-e15. https://doi.org/10.1016/j.nedt.2015.01.019


**Received:** February 21, 2021
**Accepted:** August 4, 2021
**Published:** November 8, 2021

Copyright © 2021 Lloyd et al. This is an open-access publication distributed under the terms of the Creative Commons Attribution license.
