# Supplementary material for: Addressing Trauma and Building Resilience in Children and Families: Standardized Patient Cases for Pediatric Residents
Source: MedEdPORTAL. 2021 Nov 8;17:11193. doi: 10.15766/mep_2374-8265.11193 (PMC8592119; doi:10.15766/mep_2374-8265.11193)
Supplement: Supplementary file 1 — Case 1.docxCase 2.docxCase 3.docxResource Packet.docxOrientation Slides.pptxWays to Ask About Trauma.mp4NCTSN Encounter Learner Handout.docxDe-escalation Strategies.mp4Scenario 1 Evaluation Checklist.docxScenario 2 Evaluation Checklist.docxScenario 3 Evaluation Checklist.docxDebrief Instructions.docxPresurvey.docxPostsurvey.docxEncounter-Specific Survey.docx [file mep_2374-8265.11193-s001.zip › A. Case 1.docx]

| **Appendix A: Somatic Symptoms in an Adolescent with ACEs**  **Standardized Patient** **Case**    **STANDARDIZED PATIENT** **CASE TITLE:** Adolescent with Abdominal Pain Affected by Adverse Childhood Experiences  **AUTHORS:** Jaime La Charite MD MPH  **LEARNER AUDIENCE:** Pediatric residents | |
| --- | --- |
| **PATIENT NAME:** Mariana Lopez    **PATIENT AGE:** 16 years old    **CHIEF COMPLAINT:** Abdominal pain    **PHYSICAL SETTING:** Ambulatory clinic | |
|  | |
| **Brief narrative description of case** | Learning to counsel an adolescent patient with somatic symptoms (abdominal pain) who has also been affected by trauma. |
| **Primary Learning Objectives** | 1. Inquire about stressors and exposure to trauma using neutral, non-threatening language 2. Use trauma-informed principles to create a physically and emotionally safe clinical space 3. Educate about how trauma affects physical and emotional health and influences behavior 4. Provide developmentally appropriate guidance about routines and activities that help restore safety and control after trauma, promote supportive caregiver-child relationships, and build children’s sense of self-efficacy 5. Provide specific local resources for referrals (primary, secondary, and tertiary prevention) |
| **Critical Actions** | The learner should develop rapport with Mariana that allows them to inquire about any stressors or trauma that may be contributing to Mariana’s presentation, and offer counseling regarding next steps. Specifically, the learner should:  1. Learn more about Mariana’s abdominal pain as it might relate to stressors in her life  2. Evaluate for traumatic experiences  3. Counsel Mariana about how traumatic experiences may be impacting her health  4. Learn about the protective factors in Mariana’s life that can be harnessed to promote resilience  5. Offer strategies that Mariana may employ in her everyday life to cope with her current situation. These can be general resiliency strategies. You do not have to offer specific community resources.  6. Set a plan for follow up and wrap up the encounter |
| **Learner Preparation or Prework** | **LEARNER INSTRUCTIONS**  **Patient Information:** Mariana Lopez  **Your Role:**  You are a pediatrics resident. You are working in your continuity clinic and your next patient is an adolescent who is well known to the clinic. However, she is a new patient to you. Her chief complaint is “belly pain.”  **Situation:**  Mariana Lopez is a 16-year-old female adolescent with no significant past medical history who is presenting to clinic for abdominal pain. She is alone for this visit.  **In preparation for your clinic appointment, you reviewed her chart the night before**. You note that she came to the clinic a few days ago for abdominal pain and has had multiple visits for various types of pain in different locations over the last few years.  For her abdominal pain, she has had an extensive workup already related to her multiple presentations. A few days ago, the team performed a pelvic exam that was unremarkable, as well as a sexually transmitted infection workup (including gonorrhea, chlamydia, trichomonas, syphilis, HIV), which was all negative. CBC, ESR, CRP, CMP, Lipase, UA, celiac panel, TSH, stool studies have all been normal. An abdominal and pelvic US and CT were normal. She saw GI who diagnosed her with functional abdominal pain.  *PMH/PSH:* None  *Home Meds*: Alternates Tylenol and Motrin as needed for her pain, usually two Tylenol or two ibuprofen 2-3 days a week  *Allergies:* None  *Family Hx:* Father’s history is unknown. Mother has untreated depression and ongoing alcohol use. She has two younger siblings (age 4 and 6) that are healthy.  *Social Hx/HEADSS assessment:* She is primarily cared for by her grandmother. Her mom and her two siblings also live in the home. She goes to a local public school. She enjoys school and wants to be a writer and is looking forward to going to college. She is getting mostly Bs. She is active in choir at school. She likes to play basketball with her friends, but doesn’t get to play often since it is dangerous to be outside in her neighborhood. She stays away from drugs and alcohol. She is in a relationship and sexually active with her 17-year-old boyfriend. She immigrated to the United States from El Salvador with her mother when she was 6 years old. Her favorite activities include spending time with her grandmother and singing. She notes that she feels happiest and the least amount of stress when she is doing either of those.  *Vital Signs:* Temperature 97.3, BP 130/80, Pulse 90/minute, Respirations 14/minute, BMI > 90^th^%ile  *Exam:* She was seen for abdominal pain last week and the last documented exam including HEENT, CV, pulmonary, abdominal exam, and extremity exam was unremarkable.  **Your Task:**  Develop rapport with Mariana that allows you to inquire about any stressors or trauma that may be contributing to Mariana’s presentation, and offer counseling regarding next steps. Specifically, you should:  1. Learn more about Mariana’s abdominal pain as it might relate to stressors in her life  2. Evaluate for traumatic experiences  3. Counsel Mariana about how traumatic experiences may be impacting her health  4. Learn about the protective factors in Mariana’s life that can be harnessed to promote resilience  5. Offer strategies that Mariana may employ in her everyday life to cope with her current situation. These can be general resiliency strategies. You do not have to offer specific community resources.  6. Set a plan for follow up and wrap up the encounter  ****If the information is given to you in the prompt, you do not need to ask the history questions again (i.e. past medical, medications, etc). You do NOT need to do an exam. You have NOT met Mariana yet; please introduce yourself.** |

| **INITIAL PRESENTATION** | | | |
| --- | --- | --- | --- |
| **Initial vital signs** | Temperature: 97.3  Blood pressure: 110/75  Pulse: 90/minute  Respirations: 14/minute | | |
| **Overall Setting and Appearance** | 2 chairs arranged in a typical manner in an exam room. SP is sitting in one chair. | | |
| **Standardized participant and their roles in the room at case start** | Standardized patient (SP), preferably age 15-25; street clothes, sitting in a chair. Dialogue is ad lib based on a typical clinical encounter using the below background information as a guide | | |
| **HPI** | ***Information in bold should be volunteered by standardized patients even if not asked.** Unbolded information can be offered in response to direct or indirect questions.  **You came to clinic today because your chronic abdominal pain has worsened over the last week.** **You have had intermittent, diffuse abdominal pain since childhood. The pain is achy in nature and can range from 5-10/10 in intensity. The Tylenol and Motrin only help somewhat. The pain is worse when you are stressed, but otherwise nothing else seems to improve or exacerbate the pain. You don’t know why your pain seems worse this week.** If asked, you have not had any associated diarrhea, nausea, vomiting, nor blood in your stool. If asked, you have been experiencing sleep disturbances, weight gain (10 pounds over 2 months), poor grades in school for the last few weeks given increased stress at home, frequent crying, feeling anxious. **Luckily the pain has subsided a little bit today, but you decided to come to this appointment anyway so that you could work on figuring out the cause of your abdominal pain and ways to make it better.**  Overall, you are feeling anxious about the stressors in your life and don’t understand why no one has been able to figure out why or how to address your abdominal pain. If the learner does not gain your trust, you won’t feel ready to open up about your life. You have been to this clinic before, as recently as 1 week ago, and your complaint of abdominal pain is similar. **The learner has reviewed your medical chart and full social history assessment (HEADSS assessment) and will be coming into the room already knowing this information. However, if the learner asks you this information again in a compassionate way, you can again share any of the information about your personal life when asked.**  *Social History/HEADSS assessment*: You are primarily cared for by your grandmother. Your mom and two siblings also live in the home. You go to a local public school. You enjoy school and want to be a writer and are looking forward to going to college. You are getting usually Bs. You are active in choir at school. You like to play basketball with friends, but don’t get to play often since it is dangerous to be outside in your neighborhood. You stay away from drugs and alcohol. You are in a relationship and sexually active with your 17-year-old boyfriend and use condoms. You immigrated to the United States from El Salvador with your mother when you were 6 years old. Your favorite activities include spending time with your grandmother and singing. You feel happiest and the least amount of stress when you are doing either of these.  You have been feeling more sad, distressed, and anxious for the past few weeks because your father was deported two months ago and you fear for the rest of your family’s safety. You have heard that there have been an increasing number of immigration raids over the last few years, which has exacerbated your anxiety. You are worried that you or another one of your family members may also get caught and deported. You were young when you immigrated, and you don’t remember anything about El Salvador. You are also missing your dad and are not sure when you will get to see him again. On top of this, your mother’s depression and alcohol use has seemed to worsen since your father’s deportation. **[This stress related to immigration status is a key component of your history as it is likely the root cause of your abdominal pain].** You do not have any thoughts about hurting yourself or others. No prior suicide attempts.  *Adverse Childhood Experiences (recent traumatic experiences)*: You immigrated with your mother from El Salvador to the US when you were 6 years old to join your father and grandmother living in the U.S. However, your father is no longer living with you since the deportation two months ago. Your siblings were born in the US. The rest of your extended family are back in El Salvador. You haven’t been back since because of the violence. Both your mother and you are undocumented. Your mother is living with you and is intermittently depressed and has a problem with alcohol, which makes it hard for her to work. You tell your mom you want to go to college, but she thinks that isn’t realistic. She recommends you go to beauty school. Your grandmother is very supportive of you going to college though. No one has touched you inappropriately or physically hurt you. No drug use in the home. You sometimes go without food so your siblings can eat. It happens maybe one or twice a week. You have always had a roof over your head. You were never in foster care. Even though you speak English fluently, there are kids at school that make fun of you for your immigrant accent and heritage. No one has physically harmed or threatened you at school, but other kids are often verbally abusive. You have never had a serious medical procedure or life-threatening illness. There is constant violence around your neighborhood that prevents you from spending much time outside. You were never detained, arrested, or incarcerated. You aren’t sure if you are treated badly due to your race. Your boyfriend is very supportive and is not physically or verbally abusive.  *Relevant Review of Symptoms:* If asked, you have been experiencing sleep disturbances, weight gain (10 pounds over 2 months), poor grades in school (getting C-Ds over the last few weeks when you usually get Bs), frequent crying, feeling anxious. Most of these changes started over the last 2-3 weeks. | | |
| **Past Medical/Surgical History** | **Medications** | **Allergies** | **Family History** |
| None | Tylenol and ibuprofen (Motrin) as needed from over the counter, you take two  Tylenol or two ibuprofen 2-3 days a week | None | Your Father’s medical history is unknown. Your Mother has a history of depression and ongoing alcohol use. You have two younger half-siblings (brother age 4 and sister age 6) that are healthy. You share the same mother, but not the same father as your siblings. |
| **Physical Examination** – N/A | | | |

| **INSTRUCTOR NOTES - CHANGES AND CASE BRANCH POINTS** | |
| --- | --- |
| **Learner Action** | **Standardized Patient Reaction** |
| If, without establishing trust, the learner starts asking you the recent traumatic experiences questions right away then | Respond mostly with “Why does this matter?” or “Why do you want to know?” or “Why are you asking me this?” to the questions. |
| If the learner tries to gain trust by connecting with you first and gradually starts asking more personal questions about trauma, then | Be more accepting and open to answering his/her questions. |
| The learner may open by asking about your interests, abdominal pain, or by restating some aspects of the HEADSS assessment. If the learner asks something like, “Has anything scary or upsetting happened to you recently?” or “Have there been experiences that you feel have impacted your health?” then | Bring up how you have been feeling more sad, distressed and anxious for the past few weeks since your father’s deportation and the increasing number of raids. You are worried that you or one of your family members may also get caught and deported. Your mom’s depression and alcohol use also seems worse since your father’s deportation. |
| If you are feeling worried to disclose information because you are afraid that it won’t be confidential and the learner has not already addressed confidentiality, then | Say, “I’m afraid what I might say won’t be kept private.” |
| If the learner still has not explained confidentiality, then | Ask, “Will this conversation be kept confidential?” |
| If the learner explains confidentiality including that the conversation will be confidential unless it is disclosed that you are at risk of being harmed or harming yourself, then | Say something indicating this is not a concern and you understand like, “Oh, no nothing like that... but ok, I guess I could tell you more about my family.” |
| If the learner doesn’t address how questions about trauma are related to your abdominal pain, ask, | “You are asking me a lot of personal questions. What does this have to do with my belly pain?” |
| If the learner goes on to say something like, “It looks like you have had some difficult experiences. I am sorry that these things have happened to you. Would you mind if I ask you a few more questions about your past experiences? I think it might help me understand your belly pain better.” | Be more accepting and open to answering his/her questions. Acknowledge that the stress of being undocumented and your family situation may be causing you a lot of stress. You could see how your belly pain worsens when you are feeling more stressed. |
| If the learner, doesn’t answer your question or is not empathetic then | Be more closed off and don’t respond fully to the questions. |
| If the learner alludes to that the stress you’re experiencing may be linked to your stomach pain, then | Say, “Are you saying my pain is ‘fake’?’  Note: It is helpful to push the learners, even if they are building rapport and being compassionate, to say they might be insinuating that the pain isn’t real. This will help them practice clear language to use in their explanation of the effects of stress on the immune system and the body’s hormonal regulation. |
| If the learner says something such as, “We now understand that exposure to stressful or traumatic experiences like the ones you described may increase stress hormones that can affect your health. I am concerned that these may be contributing to your stomach pain, school problems, and weight gain,” then say | “That makes sense, but that sucks. What can I do about it?” |
| If the learner does not respond as above, then say | “I still don’t get why you are asking me these questions. My stomach still hurts.” |
| If the learner next discusses one or more of the protective strategies listed below, then say   - General protective factors (e.g. talking to someone in a supportive relationship or therapy, healthy sleep, or exercise) - Specific protective factors (e.g., relationship with grandmother or love of singing) - Strategies for stress management (e.g. breathing exercises or mindfulness) | “I think I could do some of those things. I would like to learn more. I feel like life is such a mess right now. It is good to know I am doing something right.” |
| If the learner doesn’t really name specific coping strategies for you to employ at home, then respond | “Luckily my mom knows people in the community that can help and is talking to a social worker, but I just feel terrible and I am so stressed. I wish there was something I could do to help how I feel and my stomach pain.” |
| If the learner discusses their concern that you seem depressed and would like to offer a psychiatric referral, then say, | “I guess I have been feeling more down lately, but I am just stressed with everything going on. I don’t want to hurt myself or anything, but I guess it wouldn’t hurt to talk to someone about how I have been feeling.” |
|  | Thank learner |
| Simulation ends with learner leaving the room | Simulation ends |
