# Supplementary material for: Addressing Trauma and Building Resilience in Children and Families: Standardized Patient Cases for Pediatric Residents
Source: MedEdPORTAL. 2021 Nov 8;17:11193. doi: 10.15766/mep_2374-8265.11193 (PMC8592119; doi:10.15766/mep_2374-8265.11193)
Supplement: Supplementary file 1 — Case 1.docxCase 2.docxCase 3.docxResource Packet.docxOrientation Slides.pptxWays to Ask About Trauma.mp4NCTSN Encounter Learner Handout.docxDe-escalation Strategies.mp4Scenario 1 Evaluation Checklist.docxScenario 2 Evaluation Checklist.docxScenario 3 Evaluation Checklist.docxDebrief Instructions.docxPresurvey.docxPostsurvey.docxEncounter-Specific Survey.docx [file mep_2374-8265.11193-s001.zip › B. Case 2.docx]

| **Appendix B: ACE Disclosure by Parent Standardized Patient Case**    **STANDARDIZED PATIENT CASE TITLE:** Parent during 5-year-old well child visit discloses ACE  **AUTHORS:** Robin Ortiz MD    **LEARNER AUDIENCE:** Pediatric residents | |
| --- | --- |
| **PATIENT NAME:** Ms. or Mr. Carver (mother or father of Danny)    **PATIENT AGE:** 22-40 years old    **CHIEF COMPLAINT:**Well child visit    **PHYSICAL SETTING:** Ambulatory clinic | |
|  | |
| **Brief narrative description of case** | Learning to counsel a parent who discloses a recent family trauma |
| **Primary Learning Objectives** | 1. Educate about how trauma affects physical and emotional health and influences behavior 2. Provide developmentally appropriate guidance about routines and daily activities to promote recovery from trauma (coping), strengthen parent-child relationships, and restore sense of safety 3. Provide strategies for management of emotions and effective discipline 4. Provide specific local resources for referrals (primary, secondary, and tertiary prevention) |
| **Critical Actions** | The learner should ask Danny’s mother/father if they have any further questions, and then provide appropriate counseling, resources, referrals, or interventions to address Ms./Mr. Carver’s question.  Specifically, the learner should perform these steps:  1. Ask Ms./Mr. Carver if they have any additional concerns that s/he would like to discuss.  2. Respond to Ms./Mr. Carver’s concern with appropriate counseling, focusing on both the child’s and caregiver’s wellbeing.  3. Provide resources and/or intervention as appropriate to address Ms./Mr. Carver’s concern.  4. Set a plan for follow-up with Danny and close the encounter. |
| **Learner Preparation or Prework** | **LEARNER INSTRUCTIONS**  **Patient Information:** Ms./Mr. Carver  **Your Role:**  You are a pediatric learner. You are working in your continuity clinic and have just finished a 5-year- old boy’s well child check. As he goes to get his hearing and vision screen with the medical assistant, you go into the exam room to ask the mother/father if they have any more questions or concerns and wrap up the visit.  **Situation:**  Ms./Mr. Carver is the mother/father of Danny Carver, a 5-year-old boy with whom you just completed a well child check.  Ms./Mr. Carver mentioned during the well child check some minor behavioral problems in the home such as difficulty falling asleep, and you appropriately provided education regarding sleep hygiene as a part of your age-appropriate anticipatory guidance. Otherwise, Danny was noted to be growing well, developing well, and in good health.  You stepped out to tell your Medical Assistant that Danny is ready to complete a hearing and vision screening and you will be going back in during that time to make sure Danny’s mother/father has no further questions.  Danny’s history: *PMH/PSH:* Eczema *Home Meds:* Vaseline and occasional hydrocortisone 1% as needed *Family Hx:* Father has a history of eczema. Mother has a history of depression.  *Social Hx:* Danny lives with his mother and father. You met his other parent at his last well child visit last year, as both parents are involved in Danny’s medical care. Danny’s paternal grandmother also lives nearby. Danny has no brothers or sisters. He started kindergarten this year and is doing very well according to his grade reports from his teachers. You have already asked about safety at home and any recent household stressors; Ms./Mr. Carver appeared that they may have had something to discuss with you, but then did not report anything concerning.  **Your Task:**  Ask Danny’s mother/father if they have any further questions. Provide appropriate counseling, resources, referrals, or interventions to address Ms./Mr. Carver’s question.  Specifically, you should perform these steps:  1. Ask Ms./Mr. Carver if they have any additional concerns that they would like to discuss.  2. Respond to Ms./Mr. Carver’s concern with appropriate counseling, focusing on both the child’s and caregiver’s wellbeing.  3. Provide resources and/or intervention as appropriate to address Ms./Mr. Carver’s concern.  4. Set a plan for follow-up with Danny and close the encounter. |

| **INITIAL PRESENTATION** | | | |
| --- | --- | --- | --- |
| **Initial vital signs** | Temperature 97.3, BP 110/75, Pulse 90/minute, Respirations 14/minute | | |
| **Overall Setting and Appearance** | 2 chairs arranged in a typical manner in an exam room. 2 chairs facing each other in the vicinity of the center of the room. SP is sitting in one chair. | | |
| **Standardized participants and their roles in the room at case start** | Standardized patient (SP), preferably age 22-40 years; street clothes, sitting in a chair. At the beginning of the encounter, after the learner asks if you have any more questions, you say, “Well actually, there have been some things going on in our family that I’m worried may be affecting Danny.” Dialogue is then ad lib based on a typical clinical encounter using the below background information as a guide. | | |
| **HPI** | ***Information in bold should be volunteered by standardized patients even if not asked.** Unbolded information can be offered in response to direct or indirect questions.  *Background:* You, Mr./Ms. Carter, are feeling anxious about talking to Danny about his parent becoming incarcerated. You are overwhelmed with learning how to be a single working parent while missing your significant other and are not used to talking about feelings. You are feeling a loss of guidance on where to go for help or what to do.  *Your emotional state:* Currently, you are feeling more down than usual due to this recent change in circumstances, but you do not feel as hopeless as you have in the past and are not currently suicidal. It is fine for you to express your low mood in your body language or statements, as this will help the learner think about how to support caregivers as well as children during times of stress. However, you should not act so depressed as to make your mental health the main focus of the scenario.  You came to clinic today to bring your 5-year-old son, Danny Carver, for his well child visit. You’ve gone through the visit with both Danny and the learner in the room, and now Danny has gone with the medical assistant to get his hearing and vision screening done in the other room. The learner has stepped out to get your closing paperwork for the visit. You are waiting in the exam room for the provider to return to end the visit.  You live with Danny and Danny’s mother/father. You and Danny’s mother/father are not married, but have lived together for 7 years, 5 of them with Danny. You have a healthy and happy relationship. You work for Amazon in the stocking department. Danny’s mother/father has stayed home caring for him, though had plans to get a job when Danny started kindergarten.  **Three months ago, Danny’s mother/father was arrested on the street and was charged with possession with intent to distribute marijuana.**  T**hey were sentenced to 3 years in prison.** This took both you and Danny’s mother/father by complete surprise. You miss them and simultaneously have had to adjust to being a working single parent. You have had depression in the past. Currently, you are feeling more down than usual due to this recent change in circumstances, but you do not feel as hopeless as you have in the past and are not currently suicidal. Fortunately, your mother (Danny’s grandmother) lives nearby and has offered to care for Danny when you are working and Danny is not at school.  **Since all of this adjustment happened so quickly, you have not told Danny about where his mother/father is.** You haven’t decided yet whether you should tell him at all or if you would want Danny to visit his mother/father in prison.  Danny thinks that they went to spend some time with their family who live out of state and will be returning soon. You suspect that some of the difficulty sleeping that Danny is newly displaying is related to his missing his mother/father, and are afraid to talk to him about it because you don’t know what to say and are afraid to tell Danny the truth. Danny has also had a few emotional outbursts in the last week over minor mealtime issues (not wanting to eat peas) – something that never would have escalated so far in the past – and you wonder if this could be related to everything going on at home. You haven’t told the learner about this yet.  *Additional Social Hx:* The learner met Danny’s mother/father at his last well child visit last year, as both of you are involved in Danny’s medical care. Danny has no brothers or sisters. He started kindergarten this year and is doing very well according to his grade reports from his teachers.  *Adverse Childhood Experiences (previous traumatic experiences)*: Your father left when you were about Danny’s age.  *Relevant Review of Symptoms:* Negative  You hope that, since Danny is not in the room, you might be able to ask the learner **if you should tell Danny and how to do so**. You also hope to get resources on how to help Danny so he won't be agitated anymore. | | |
| **Past Medical/Surgical History** | **Medications** | **Allergies** | **Family History** |
| None | Vaseline and occasional hydrocortisone 1% as needed | None | Father has a history of eczema. Mother has a history of depression. |
| **Physical Examination** – N/A | | | |

| **INSTRUCTOR NOTES - CHANGES AND CASE BRANCH POINTS** | |
| --- | --- |
| **Learner Action** | **Standardized Patient Reaction** |
| Knock and enter; ask Danny’s mother/father if s/he has any more concerns | “Well actually, there have been some things going on in our family that I’m worried may be affecting Danny.” |
| The learner should ask an open-ended question for you to explain further | Unfortunately, and unexpectedly, Danny’s mother/father was recently sentenced to 3 years in prison and I haven’t told Danny. |
| If the learner DOES NOT ask an open-ended question, then | Answer only yes or no until the learner asks an open-ended question, and when the learner does, respond as above  Note: The information about Danny’s parent being incarcerated is critical information and should be disclosed in some way even if the learner does not initially start with an open-ended question as this is the basis for the encounter |
| The learner should then comfort or reassure you and ask a follow-up question about the situation with Danny. For example: “That must be a difficult situation for you and Danny. I’m sorry to hear about this. Could you tell me more about your thoughts in not sharing this with Danny?” If they ask a question of this nature, then | Describe why you have not communicated this with Danny explaining your fear of his view of his mother/father and how he will react when he hears the news |
| If the learner asks you how you are feeling (not required), then respond: | “I am feeling pretty down about it, and stressed out, but I have family support and I’m just wondering how this is going to affect Danny.” |
| If the learner asks you how they can best support you or whether you are interested in mental health resources, say | “I’m willing to hear about anything that you think will help Danny and me get through this.” |
| If the learner asks you why Danny’s parent is incarcerated | You can be forthcoming with the reason of Danny’s parent’s incarceration like, “He was found guilty of intent to distribute marijuana,”  Note: This is NOT essential information to share in the encounter for the learner to meet the encounter goals, but if there is an opportunity to divulge the information, please do so easily as it may allow the learner to practice responding non-judgmentally |
| If the learner responds non-judgmentally, then | Thank the learner |
| If the learner responds in a way that is perceived as judgmental, then | Close up a bit non-verbally such as crossing your arms and say something like, “You know that’s not a big deal, it really wasn’t his fault and it’s people responding like that, that makes me not want to share things with Danny.” |
| If you are feeling worried to disclose information because you are afraid that it won’t be confidential and the learner has not already addressed confidentiality, then | Say, “I’m afraid what I might say won’t be kept private.” |
| If the learner still has not explained confidentiality, then | Ask, “Will this conversation be kept confidential?” |
| If the learner explains confidentiality including that the conversation will be confidential unless it is disclosed that Danny is at risk of being harmed by himself or someone else, then | Say something indicating this is not a concern and you understand like, “Oh no, Danny and I feel very safe at home. Thank you for clarifying that.” |
| If the learner does not ask more about the communication with Danny and starts talking in general about traumatic childhood experiences, you should: | Gently interrupt or respond with something like, “Wait, wait a second. I want to know if I should even talk to Danny about this and how.” |
| The learner should at some point invite conversation about physical or behavioral symptoms that Danny may be having related to this new family change. If the learner asks about Danny’s difficulty sleeping, or if Danny has had any other symptoms that you think might be related, then | Agree that sleeping problems may be related to mom/dad’s recent incarceration. Also disclose Danny’s recent emotional outbursts over food issues at mealtimes |
| If the learner never brings up physical or behavioral symptoms, then | Ask whether Danny’s sleeping problems or outbursts could be related to parent’s incarceration |
| In either scenario above, the learner should then counsel you on the importance of communication and honesty. S/he may provide resources like a tool or game for practicing emotion sharing and communication, or referral counseling, or a handout from Sesame Street Communities on Coping with Incarceration. | If the learner has provided these tools, you should thank them. |
| If the learner has not presented resources on communication, but explained the importance of sharing this information with Danny, you should, | Ask, "Ok, I understand I should tell him, but how? Are there resources or people that could help?” |
| If the learner offers to help you disclose this news to Danny today in the office, then say | “I think I would rather tell him at home in his room, where he feels safe. Do you think that would be okay?” Learner should answer, “Yes,” and offer to be a resource any time in the future. |
| The learner should then explain that resilience can be built after exposure to an adversity like separation from a parent through incarceration and offer strategies such as: establishing and maintaining old routines at home, as well as creating new routines and traditions; remaining calm during outbursts; naming emotions; spending dedicated parent-child time every day; positive parenting approaches, etc.  Note: it is not expected for them to mention all of these or any specifics, but they should offer some description of how the parent-child relationship and emotional intelligence can build resilience. | If the learner does this, you can thank them for being encouraging and providing resources and ask when you should return to follow-up. |
| If the learner provides resources with little explanation, you should: | Ask more questions about the materials, how to talk to Danny using them, to prompt the learner to share more information. |
| If the learner asks you about how you are coping with the loss of your partner, say | “It’s been really hard, and I think it’s made my depression even worse than they already were.” |
| If the learner asks you if you would be interested in a referral to mental health resources, then | Accept any resources that they can provide |
| The encounter ends with the parent thanking the learner and the learner leaving the room for their next patient. |  |
