# Supplementary material for: Addressing Trauma and Building Resilience in Children and Families: Standardized Patient Cases for Pediatric Residents
Source: MedEdPORTAL. 2021 Nov 8;17:11193. doi: 10.15766/mep_2374-8265.11193 (PMC8592119; doi:10.15766/mep_2374-8265.11193)
Supplement: Supplementary file 1 — Case 1.docxCase 2.docxCase 3.docxResource Packet.docxOrientation Slides.pptxWays to Ask About Trauma.mp4NCTSN Encounter Learner Handout.docxDe-escalation Strategies.mp4Scenario 1 Evaluation Checklist.docxScenario 2 Evaluation Checklist.docxScenario 3 Evaluation Checklist.docxDebrief Instructions.docxPresurvey.docxPostsurvey.docxEncounter-Specific Survey.docx [file mep_2374-8265.11193-s001.zip › C. Case 3.docx]

| **Appendix C: De-escalation** **Standardized Patient Case**    **STANDARDIZED PATIENT CASE TITLE:** De-escalation of an Adolescent Affected by Adverse Childhood Experiences    **AUTHOR:** Jaime La Charite MD MPH    **LEARNER AUDIENCE:** Pediatric residents | |
| --- | --- |
| **PATIENT NAME:** Jordan Wilcox    **PATIENT AGE:** 16    **CHIEF COMPLAINT:** Aggression at school    **PHYSICAL SETTING:** Emergency room | |
|  | |
| **Brief narrative description of case** | Learning trauma-informed de-escalation techniques |
| **Primary Learning Objectives** | 1. Remain safe during the clinical encounter 2. Demonstrate de-escalation techniques, inclusive of noticing signs of distress, connecting with youth, redirection, discussion about what happened once calm, trauma-informed consequences |
| **Critical Actions** | The learner should utilize de-escalation techniques to develop a therapeutic relationship with Jordan. They should consider trauma and use trauma-informed care to engage with, evaluate, and create a care plan for this patient.  In particular, the learner should perform these steps:  1. Position themselves safely in the room and ensure safe conditions for de-escalation.  2. Use verbal & non-verbal skills for de-escalation **(This is the primary goal of the case)**  3. Use trauma informed strategies to evaluate why Jordan had an outburst at school.  4. Provide Jordan with resiliency strategies to help manage strong emotions. The focus should be on strategies to use in the future, rather than correcting the behavior witnessed today. It is OK for this final step to remain unresolved if there is insufficient time, as the primary goal of the encounter is to help de-escalate the patient. |
| **Learner Preparation or Prework** | **LEARNER INSTRUCTIONS**  **Patient Information:** Jordan Wilcox  **Your Role:**  You are working in the pediatric emergency room. You are assigned to care for an adolescent patient that was brought in by the police after being aggressive at school. Before you get to see them , the nurse calls to tell you that they are yelling at the staff.  **Situation:**  Jordan is a 16-year-old male or female adolescent with no significant past medical history who is presenting after being brought in by police for being aggressive at school. They are alone for this visit.  You find out from the social worker that Jordan started yelling at their teacher & classmates and throwing items in their classroom. They then tried to hit one of the other students. They were recently seen for a well adolescent visit. You gather the following information from their most recent pediatrician’s note.  *PMH/PSH:* None *Home Meds:* None *Allergies:* None *Family Hx:* None. *Social Hx:* They are living with their mother and father. Their mom works two jobs so isn’t at home much. Their father is an alcoholic. Their older brother is in jail. They go to an inner-city poor-performing high school. They have been getting mostly Cs in their classes, but are in danger of dropping their grades to D & Fs. They play soccer for their school and need to maintain a C average to continue playing on the team. They smoke marijuana and drink a couple beers at parties. They are sexually active with their 16-year-old boy/girlfriend. They always use condoms. Their mood is sad sometimes.  Vital Signs: Temperature 97.3, BP 110/75, Pulse 90/minute, Respirations 14/minute  **Your Task:**  Utilize de-escalation techniques to develop a therapeutic relationship with Jordan. Consider trauma and use trauma-informed care to engage with, evaluate, and create a care plan for this patient.  In particular, you should perform these steps:  1. Position yourself safely in the room and ensure safe conditions for de-escalation. This may require you to leave the room. If you decide to leave the room, you can assume stepping outside for 1 minute is equivalent to stepping outside the room for 5-10 minutes in a real life situation.  2. Use verbal & non-verbal skills for de-escalation **(This is the primary goal of the case).**  3. Use trauma informed strategies to evaluate why Jordan had an outburst at school.  4. Provide Jordan with resiliency strategies to help manage strong emotions. Your focus should be on strategies to use in the future, rather than correcting the behavior witnessed today. It is OK for this final step to remain unresolved if there is insufficient time, as the primary goal of the encounter is to help de-escalate the patient.  ****If the information is given to you in the prompt, you do not need to ask the history questions again (i.e. past medical, medications, etc.). You do not need to do an exam. Keep in mind that is a condensed encounter. In reality, the above steps may occur over a longer period than 20 minutes.** |

| **INITIAL PRESENTATION** | | | |
| --- | --- | --- | --- |
| **Initial vital signs** | Temperature 97.3, BP 110/75, Pulse 90/minute, Respirations 14/minute | | |
| **Overall Setting and Appearance** | The room is set up as a clinical exam room. The SP is pacing around the room, agitated, at the beginning of the scenario. There are 2 chairs for the SP and learner to sit in during the case if they choose to do so. | | |
| **Standardized participants and their roles in the room at case start** | Standardized patient (SP), preferably age 15-25; street clothes, pacing around the exam room, appearing agitated. The SP bangs fists on tables and/or walls while waiting for the learner to enter. Dialogue is ad lib using the below background information as a guide | | |
| **HPI** | You are feeling agitated because you failed your test today, even though you studied. Your dad has been drinking a lot lately, and both he and classmates have been making fun of you. Specifically, you became upset today after another student whispered disrespectful comments to you in your ear during class. You started throwing objects around the room, and then tried to punch the other student. You were brought in by the police for being aggressive with teachers and students at school.  **NOTE:** The primary goal of this encounter is for the learner to practice ways to de-escalate an angry patient. There are numerous details about the story and about Jordan’s background described below that may be shared if they come up but are not essential to the goal of the encounter. **For you, the most important background to know is that the stressor that occurred today (being belittled by a classmate after failing a test despite studying hard) triggered your own reaction to prior trauma (your father being frequently drunk and belittling you at home) and led to a fight.**  If the learner doesn’t try to deescalate the situation first and gain your trust, you will become more upset and not open up about what’s been going on. The goal of this scenario is for the learner to practice de-escalation techniques. It is okay if the scenario does not reach the point where you are having a very calm and rationale conversation.  **Guidelines for escalation:** Since the goal of the encounter is the focus on the learner’s ability to help de-escalate an angry patient, you may act angry, and continue to escalate if the learner does not follow the prompts as in the chart below. For example, you may bang the table or exam table; remain standing or pacing throughout most/all of the encounter; curse or yell; or throw something on the floor like a tissue box.  **Background about learner communication in the encounter:** Remember that you are playing an adolescent. Adolescents respond best to healthy boundaries. Keep in mind that if the learner sets ground rules, this is likely because they are meeting the goals of the task and not because they are trying to talk down to you. However, if you feel they are not treating you fairly, of course, respond as such.  ***Social History***: You are living with your mother and father. Your mom works two jobs so isn’t at home much. Your father is an alcoholic and your older brother has been in jail for about a year. You go to an inner-city, poor-performing high school and have been getting mostly Cs in classes with an occasional D. You play soccer for your high school. If your grades get any worse, you are at risk for getting suspended from the soccer team. You smoke marijuana a few times a week and drink a couple beers at parties with friends. You are sexually active with your 16-year-old boy/girlfriend and have had 2 other partners since age 14. You always use condoms. Your mood is sad sometimes.  ***Adverse Childhood Experiences (previous traumatic experiences)***: Your parents are living together, but often fight at home. Your dad is an alcoholic, but no other mental illness in the family. Your dad can become verbally abusive with you and your mom when he drinks. You have not experienced physical or sexual abuse. Your dad lost his job a couple months ago due to his drinking. Your mom is very supportive, but she has to work a lot so isn’t around much. Your dad doesn’t think you will make much of your life and thinks you will end up in jail like your older brother. While sometimes finances can be tight, you always have enough food, the utilities have never been shut off, and you have always had a roof over your head. You were never in foster care. There are kids at school that make fun of your dad for being a “drunk.” You have never been physically harmed or threatened by the kids at school, but often hear derogatory comments about your dad or your academic failures. You have never had a serious medical procedure or life-threatening illness. There is constant violence around your neighborhood that prevents you from spending much time outside, so you spend a lot of time inside watching TV and movies. You were never detained, arrested, or incarcerated. You aren’t sure if you are treated badly due to your race. Your boy/girlfriend is very supportive and is not physically or verbally abusive.  **Relevant Review of Symptoms:** You have been experiencing sleep disturbances and anxiety for the past few months. | | |
| **Past Medical/Surgical History** | **Medications** | **Allergies** | **Family History** |
| None | None | None | None |
| **Physical Examination** **–** N/A | | | |

| **INSTRUCTOR NOTES - CHANGES AND CASE BRANCH POINTS**  **Opening Statement:** “Get out of here! I don’t want to talk to anyone!” SP is pacing around the room, appearing frustrated. | |
| --- | --- |
| **Learner Action** | **Standardized Patient Reaction** |
| Speaks in a calm voice. “I can see that you are upset and understand you do not want to talk to anyone right now. How about I step out of your room and give you some time to cool off in a safe place?” | Nod your head yes |
| If the learner tries to attempt to question you or lecture you about being disrespectful rather than giving you a cool off period, then respond | “I don’t want to talk to you! Get out!” and then stop talking. Continue to pace and shut down until the learner leaves the room. |
| When the learner returns, “Hello. My name is [blank], I am a [blank]. I am a doctor, my job is to try to help. What do you like to be called?” | You are now calmer, but still a little visibly agitated. “My name is Jordan. How are you supposed to help me?!” |
| If the learner doesn’t introduce themselves, then respond | “Who are you anyway? What do all you people want? I just want to go home!” |
| If the resident asks permission to continue the conversation, for example “Jordan, may I share some of my thoughts?”, then respond | “If you have to, this whole thing is dumb.” |
| If the learner threatens to give medication or discharge you if the behavior continues then respond... | “This is ridiculous! Just let me leave then...” |
| If the learner sets ground rules such as saying, “I want to help you, but in order to do that, we both need to create a feeling of safe space such as agreeing not to throw things or hit things. Does that make sense?” | Then you should respond by agreeing with a nod and an “Ok, I guess”, for example. |
| If the learner says something non-judgmental about what happened today, such as “Thank you for talking with me. I do ask that you remain calm so that we can keep talking. You seem like you are feeling angry and frustrated. Tell me if I have this right, you had a hard day at school and having a hard time cooling off. Does that sound about right?”, then respond | “Yeah, I guess so. It has been happening a lot lately.” |
| If the learner starts lecturing, telling you how to act, or not being empathetic, then respond, | “I don’t like how you’re are talking to me. I want to talk to someone else or be left alone.” |
| If you are feeling worried to disclose information because you are afraid that it won’t be confidential and the learner has not already addressed confidentiality, then | Respond “Why should I tell you anything? You probably just want to report me anyway.” |
| If the learner still has not explained confidentiality, then | Ask, “Well is there any privacy to what I say or are you just gonna go tell the world my problems?” |
| If the learner explains confidentiality including that the conversation will be confidential unless it is disclosed that you are at risk of harming yourself or someone else, then | Say something indicating this is not a concern like, “I don’t want to hurt myself or anyone, I just feel so pissed off all the time!” |
| If the learner asks what happened at school, then respond | “I took a swing at another kid. But the teacher wasn’t paying attention to the fact that this kid was saying things in my ear, so I just blew up at him.” |
| If the learner tries to help you identify the emotions you were feeling or why you reacted the way you did, for example by asking “Can you tell me what you thought about when the kid was talking in your ear?” or by stating, “It sounds like what that kid said to you made you feel really frustrated and angry” then respond | “Yeah, it gets me so pissed off because people just won’t lay off. I feel like I can’t do anything right.” |
| If the learner uses reflective listening and asks you more, for example by saying “That’s tough. Do you want to talk about what has been going on?” then | Reveal more information, for example by saying “My dad is a jerk when he drinks. He has been drinking a lot lately. Whenever he drinks he tells me I’m an idiot and will end up like my brother in jail. Even though I studied hard, I failed my test today and the other kids in class started making fun of me just like my old man dad. I couldn’t take it anymore.” |
| If the learner uses reflective listening and asks more about other ways of reacting, for example by saying, “I am sorry about your dad. I can see why what happened at school could remind you of the stuff at home be a trigger for you. I wonder if there are other ways you could respond in those types of situations at school,” then | Say “I don’t know... I have a hard time controlling my anger when something sets me off.” |
| If the learner starts lecturing you about how those experiences don’t give you a reason to be disrespectful, then respond | “I really don’t want to talk about this.” Then become more closed off and give only short yes/no answers to questions. |
| If the learner discussing coping strategies to manage strong emotions, for example by saying, “So it seems like when you get upset you have a hard time figuring out how to manage that energy. This is very common. Youth who have experienced trauma in their life can have triggers that remind them of their prior trauma, which causes them to react strongly. It sounds like you already recognize some triggers for you, which is a great first step.  The next step is to think about how to respond to these triggers differently and how to manage strong emotions. You can try talking to a trusted friend/caring adult, paying attention to what is happening in your body and taking deep breaths or using a stress ball, take a walk/run, try writing/drawing, meditate, get involved in church community, volunteer, mentor. Do any of these sound like they could help?” then | Respond “Yeah, maybe. I like to draw and my English teacher has been supportive in listening to what has been happening in my life.” |
| If the learner doesn’t provide any ideas or provides ideas that are very abstract (like getting involved in the community or finding a mentor) then respond, | “I still don’t know what to do when I get angry. I am worried I am going to end up in jail like my brother, just like my dad says.” |
| The learner should ideally respond: “That sounds like a great plan. It is good that you have some resources and activities that you can think about to help manage your emotions in these situations”. | Thank learner |
| Simulation ends with learner leaving the room | Simulation ends |

**Ideal Scenario Flow**

 The learners enter the room to find an agitated, escalated adolescent patient. The SP uses verbal and nonverbal communication to convey emotional distress. The learner may leave the room for a few minutes to allow a “cool off” period, then reenter. Over the first half of the encounter, the SP remains angry and frustrated, allowing the learner to practice de-escalation techniques. If the learner is effective at de-escalation, the second half of the encounter is spent in calmer dialogue, with the learner discussing ways to deal with future stressors/triggers. The main focus of the scenario is de-escalation; therefore it is acceptable to spend most or all of the encounter upset if the learner is not demonstrating effective de-escalation techniques.

**Anticipated Management Mistakes**

1. Difficulty with acting out agitation/frustration: We found that the SPs were initially not acting out agitation or escalation to the degree that was needed, or for the duration that was needed, for the learners to practice de-escalation techniques. Performance improved after we discussed specific techniques for demonstrating agitation and clarified the need for heightened emotion during the encounters.
