# Supplementary material for: Addressing Trauma and Building Resilience in Children and Families: Standardized Patient Cases for Pediatric Residents
Source: MedEdPORTAL. 2021 Nov 8;17:11193. doi: 10.15766/mep_2374-8265.11193 (PMC8592119; doi:10.15766/mep_2374-8265.11193)
Supplement: Supplementary file 1 — Case 1.docxCase 2.docxCase 3.docxResource Packet.docxOrientation Slides.pptxWays to Ask About Trauma.mp4NCTSN Encounter Learner Handout.docxDe-escalation Strategies.mp4Scenario 1 Evaluation Checklist.docxScenario 2 Evaluation Checklist.docxScenario 3 Evaluation Checklist.docxDebrief Instructions.docxPresurvey.docxPostsurvey.docxEncounter-Specific Survey.docx [file mep_2374-8265.11193-s001.zip › D. Resource Packet.docx]

**Resource Packet and Pre-Reading for Trauma & Resilience Standardized Patient Session**

1. American Academy of Pediatrics. Trauma Toolbox for Primary Care: The Medical Home Approach to Identifying and Responding to Exposure to Trauma. <https://www.aap.org/en-us/advocacy-and-policy/aap-health-initiatives/healthy-foster-care-america/Pages/Trauma-Guide.aspx#trauma>. Published 2014. Accessed October 31, 2020. [NOT FOR PUBLICATION]
2. Center on the Developing Child. The Science of Resilience(InBrief). [www.developingchild.harvard.ed](https://developingchild.harvard.edu/)u. Published 2015. Accessed October 31, 2020. [NOT FOR PUBLICATION]
3. De-escalation Tips. Adapted from: Richmond JS, et al. Verbal de-escalation of the agitated patient: Consensus statement of the American Association for Emergency Psychiatry Project BETA De-Escalation Workgroup. West J Emerg M. 2012; 13(1):17-25.
4. Optional List of Resources for Building Resilience

Note: Items 3 and 4 are included in the subsequent pages of this packet.

**De-escalation Tips:**

*Adapted from Richmond et al. “Verbal de-escalation of the agitated patient: Consensus statement of the American Association for Emergency Psychiatry Project BETA De-Escalation Workgroup*.” *Available at* [*https://www.ncbi.nlm.nih.gov/pmc/articles/PMC3298202/*](https://www.ncbi.nlm.nih.gov/pmc/articles/PMC3298202/)

**Remember, it’s not about you:**

- Put on your trauma lens
- Try not to take statements personally or respond defensively
- Find something to agree with or even apologize for, even if you feel the patient has an incorrect understanding of the situation
- Look for opportunities to help the patient regain a sense of efficacy and control

1. **Respect personal space and maintain safety:** at least 2 arms’ lengths between yourself and the patient, and easy proximity to an exit.
2. **Do not be provocative:** use open body language (hands visible, arms not folded, standing at an angle so as not to appear confrontational, eye contact but not excessively so).
3. **Establish verbal contact:** ensure only 1 person verbally interacts with patient at a time. Introduce yourself and your role.
4. **Listen closely to what the patient is saying:** use active and open listening (“Tell me if I have this right…”).
5. **Be concise:** keep it simple as agitated patients may be impaired in ability to process verbal information. Use language like, “I see that you are upset. I am glad you are able to share how you are feeling.” Keep your voice low and calm. Use repetition to help with processing.
6. **Identify wants and feelings:** ask the patient what their request is, even if it cannot be granted (“Even if it can’t be provided, I would like to know so we can work on it”). Use body language to identify and name feelings (“You seem like you are feeling angry and frustrated”).
7. **Agree and/or validate:** try hard find a way to agree with or validate some aspect(s) of what the patient is saying.
8. **Set clear limits:** relate your desire to help to the need to establish safe, basic working conditions: "I can tell how upset you are, but to be able to communicate with you I am going to ask that you not raise your voice or use inappropriate language. I am here to help you and address your concerns, but it hard when you are this upset. I am going to let you have some time to calm down and I will be back in 15 minutes to continue this conversation.”
9. **Coach patient to stay in control:** once you’ve established a relationship, provide verbal guidance to help the patient stay in control: “I really want you to sit down; when you pace, I feel frightened, and I can't pay full attention to what you are saying. I bet you could help me understand if you were to calmly tell me your concerns.”
10. **Offer choices and optimism:** offer alternatives to violence and acts of kindness (blankets, drinks, snacks, access to a phone). However, don’t make promises that cannot be met.

***Optional: Resources for Building Resilience***

FOR YOUNG CHILDREN AND THEIR CAREGIVERS

- **Sesame Street in Communities** - <https://sesamestreetincommunities.org/topics/>

A series of toolkits, created in collaboration with the Robert Wood Johnson Foundation, for caregivers and professionals on topics including Resilience, Traumatic Experiences, and Dealing with Incarceration. Includes videos, workshops, and activities. Favorites include the “Breathe, Think, Do" App (available for Android or iPhone) as well as PDF guides with resilience activities found at [sesamestreet.org/challenges](http://sesamestreet.org/challenges).

- **Generation Mindful** – [https://genmindful.com](https://genmindful.com/)

A company that has created tools, toys, and programs to encourage self-regulation in children, classroom management, and positive parenting skills in caregivers.  A favorite includes the Time-In Toolkit, which helps create a calming space for children to process emotions.

- **Mind Yeti** - <https://www.mindyeti.com/>

A web- and app-based platform with guided mindfulness exercises for many situations including study time, bed time and everything in between.

FOR TEENS AND ADULTS

- Mindfulness Apps:
  - **Calm** - <https://www.calm.com/> #1 App for mindfulness. Free trial available.
  - **Headspace** - [https://www.headspace.com](https://www.headspace.com/) Short sessions on topics ranging from stress to sleep. Includes SOS exercises in case of sudden meltdowns.
  - **SmilingMind** - <https://www.smilingmind.com.au/> Free app for young people.
- Mindfulness Websites:
  - **Mindful** - <https://www.mindful.org/audio-resources-for-mindfulness-meditation/> Free guided meditation audio of various lengths.
  - **The Mindful Teen** - <http://mindfulnessforteens.com/> A guide for teens to discover their inner strength.  Website includes education on mindfulness as well as a number of free guided meditations.
- **PTSD Coach** - <https://mobile.va.gov/app/ptsd-coach>

This app provides education about Post-Traumatic Stress Disorder (PTSD), information about professional care, a self-assessment for PTSD, opportunities to find support, and tools that can help you manage the stresses of daily life with PTSD symptoms.

- **Anxiety Canada** - <https://www.anxietycanada.com>

A website with information about anxiety in children, teens, and adults, including many PDFs with management strategies including breathing exercises, progressive muscle relaxation, and grounding techniques.

- **Affordable Yoga Resources**

Hundreds of channels on YouTube including Gaiam Yoga, Yoga for Dummies, DoYogaWithMe.  Or check out a “Community Class” in your local community. Example in Baltimore: YogaWorks Baltimore or Baltimore Yoga Village!

- **The Shared Grief Project**  - [http://www.sharedgrief.org](http://www.sharedgrief.org/)

Shares powerful videos from famous individuals who have experienced a major loss at an early age who have gone on to live healthy, happy, and successful lives.

***Local Organizations that Serve Trauma-Affected Families***

*Please create a list of local organizations here to educate learners about what is available near you! You may consider including specific organizations relevant to immigration and parental incarceration given relevance to standardized patient scenarios.*
