# Supplementary material for: Addressing Trauma and Building Resilience in Children and Families: Standardized Patient Cases for Pediatric Residents
Source: MedEdPORTAL. 2021 Nov 8;17:11193. doi: 10.15766/mep_2374-8265.11193 (PMC8592119; doi:10.15766/mep_2374-8265.11193)
Supplement: Supplementary file 1 — Case 1.docxCase 2.docxCase 3.docxResource Packet.docxOrientation Slides.pptxWays to Ask About Trauma.mp4NCTSN Encounter Learner Handout.docxDe-escalation Strategies.mp4Scenario 1 Evaluation Checklist.docxScenario 2 Evaluation Checklist.docxScenario 3 Evaluation Checklist.docxDebrief Instructions.docxPresurvey.docxPostsurvey.docxEncounter-Specific Survey.docx [file mep_2374-8265.11193-s001.zip › E. Orientation Slides.pptx]

## Slide 1
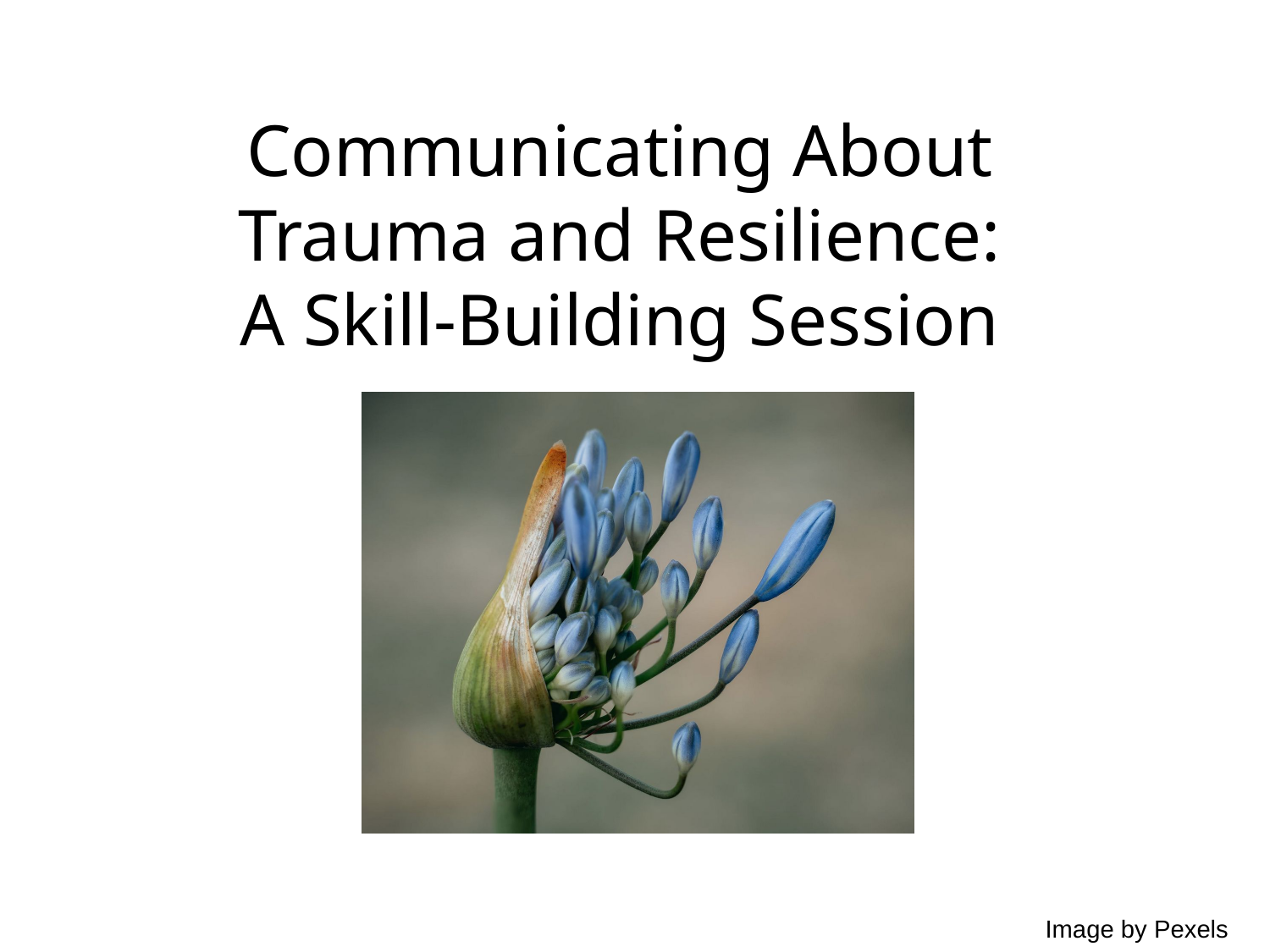

# Communicating About Trauma and Resilience: A Skill-Building Session
Image by Pexels

## Slide 2
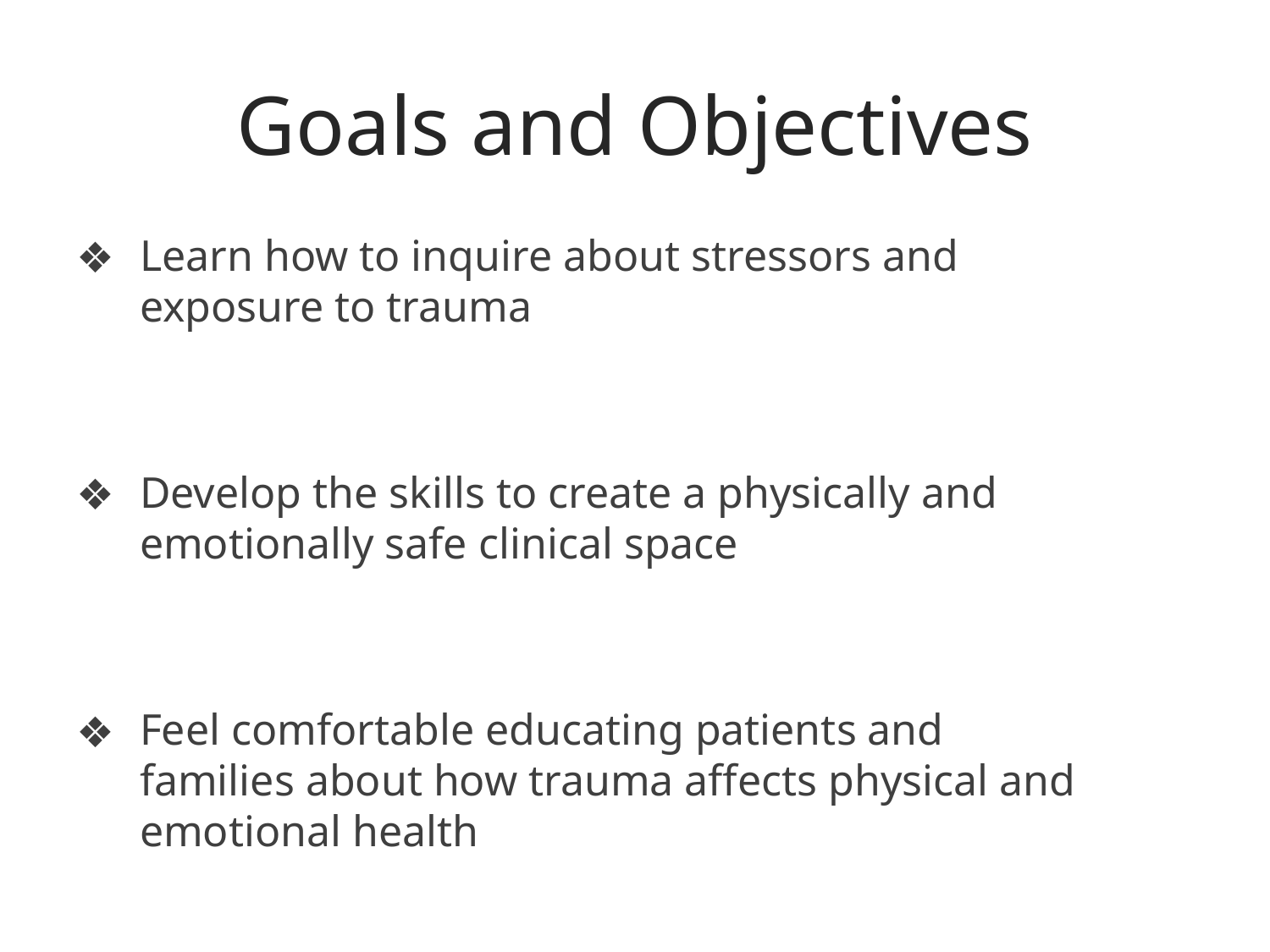

# Goals and Objectives
Learn how to inquire about stressors and exposure to trauma
Develop the skills to create a physically and emotionally safe clinical space
Feel comfortable educating patients and families about how trauma affects physical and emotional health

## Slide 3
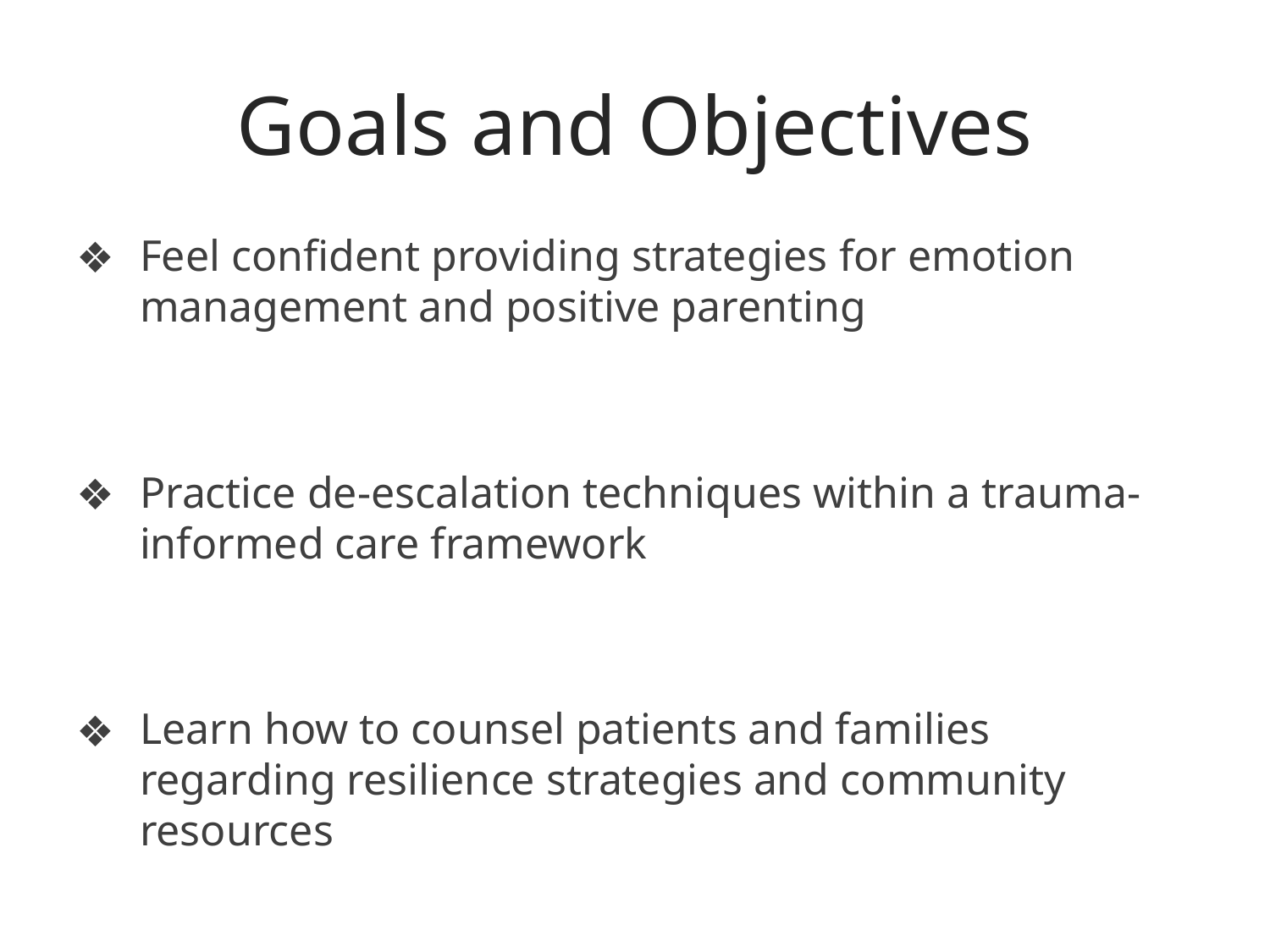

# Goals and Objectives
Feel confident providing strategies for emotion management and positive parenting
Practice de-escalation techniques within a trauma-informed care framework
Learn how to counsel patients and families regarding resilience strategies and community resources

## Slide 4
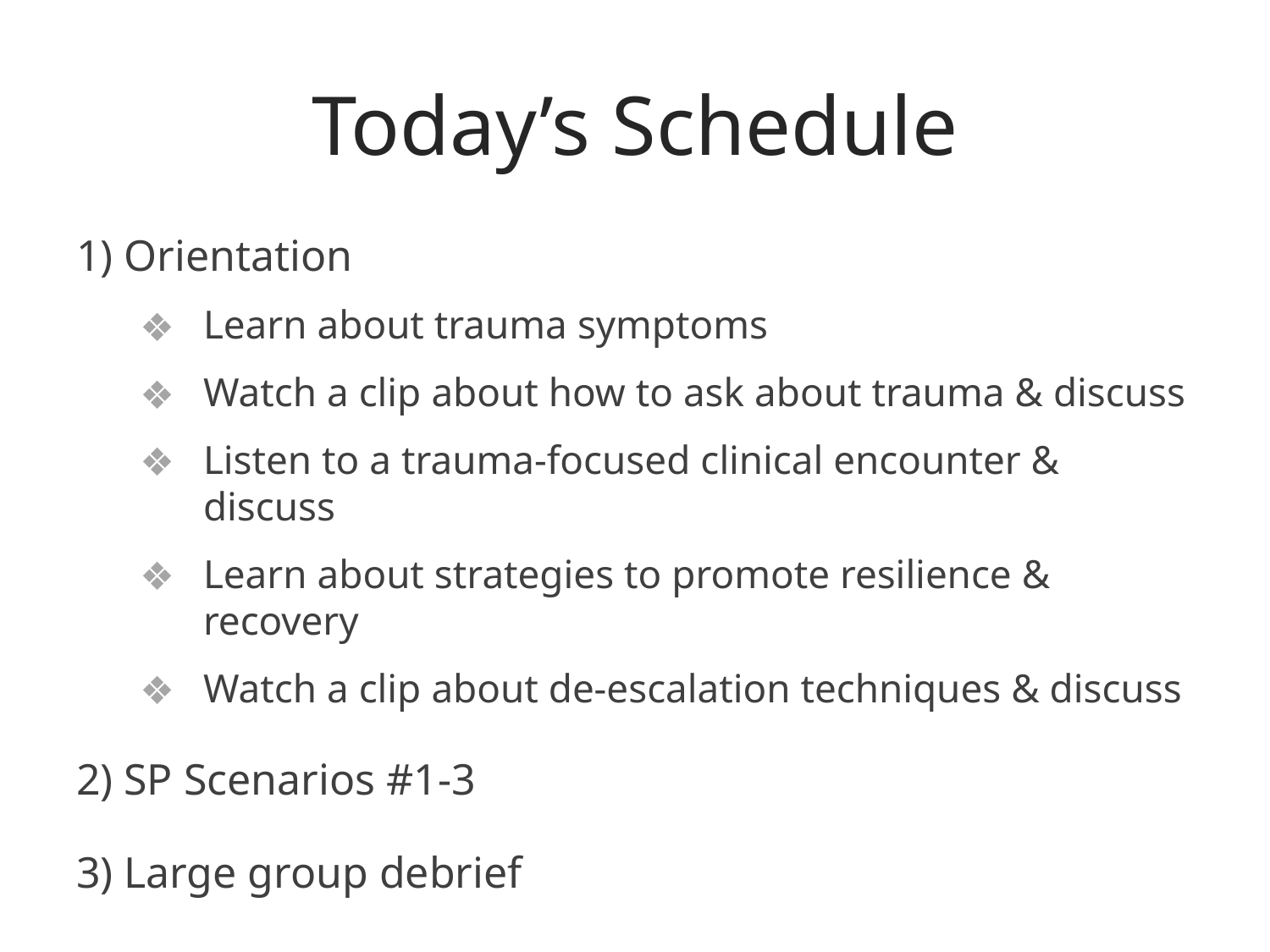

# Today’s Schedule
1) Orientation
Learn about trauma symptoms
Watch a clip about how to ask about trauma & discuss
Listen to a trauma-focused clinical encounter & discuss
Learn about strategies to promote resilience & recovery
Watch a clip about de-escalation techniques & discuss
2) SP Scenarios #1-3
3) Large group debrief

## Slide 5
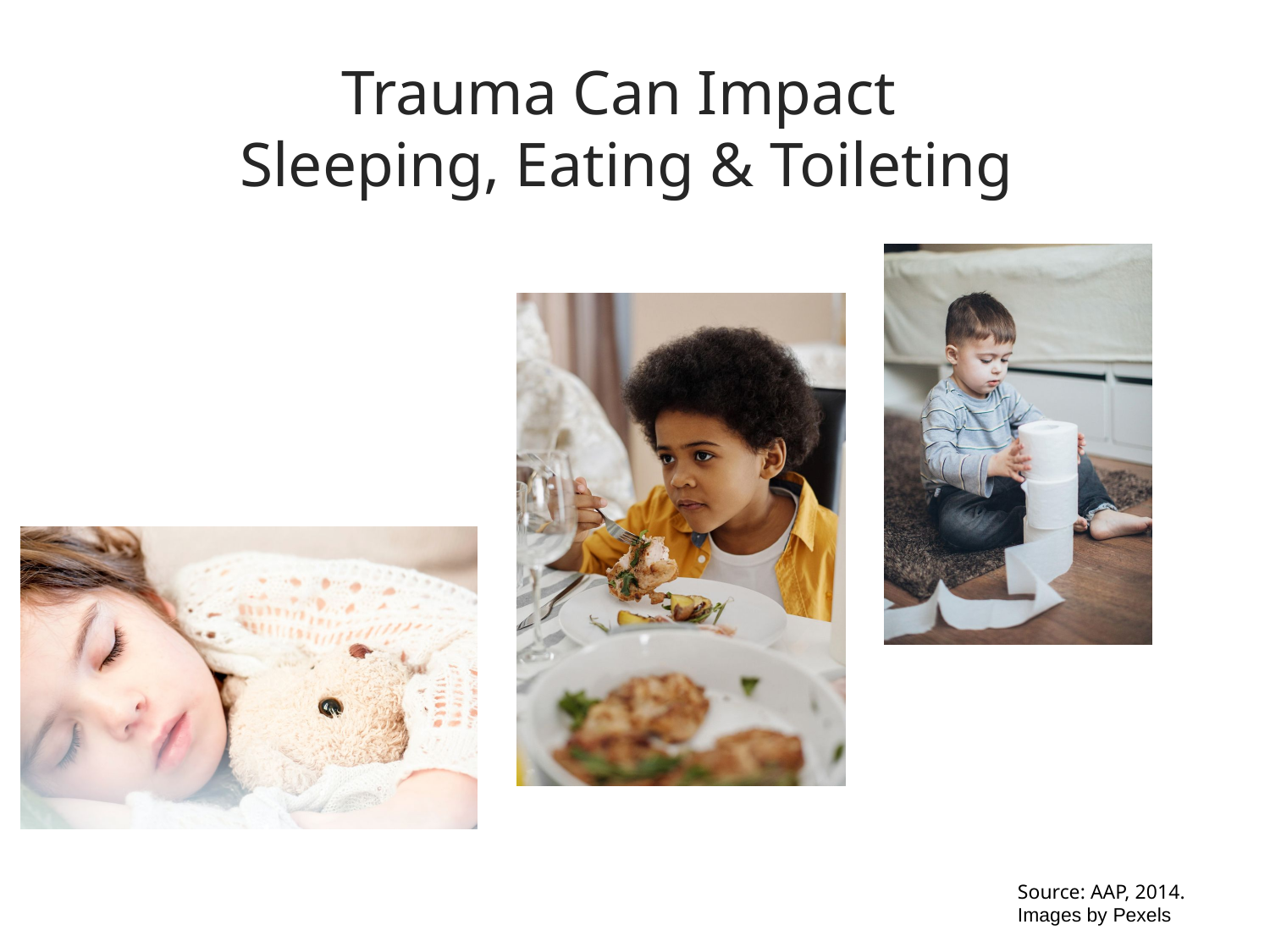

# Trauma Can Impact
Sleeping, Eating & Toileting
Source: AAP, 2014.
Images by Pexels

## Slide 6
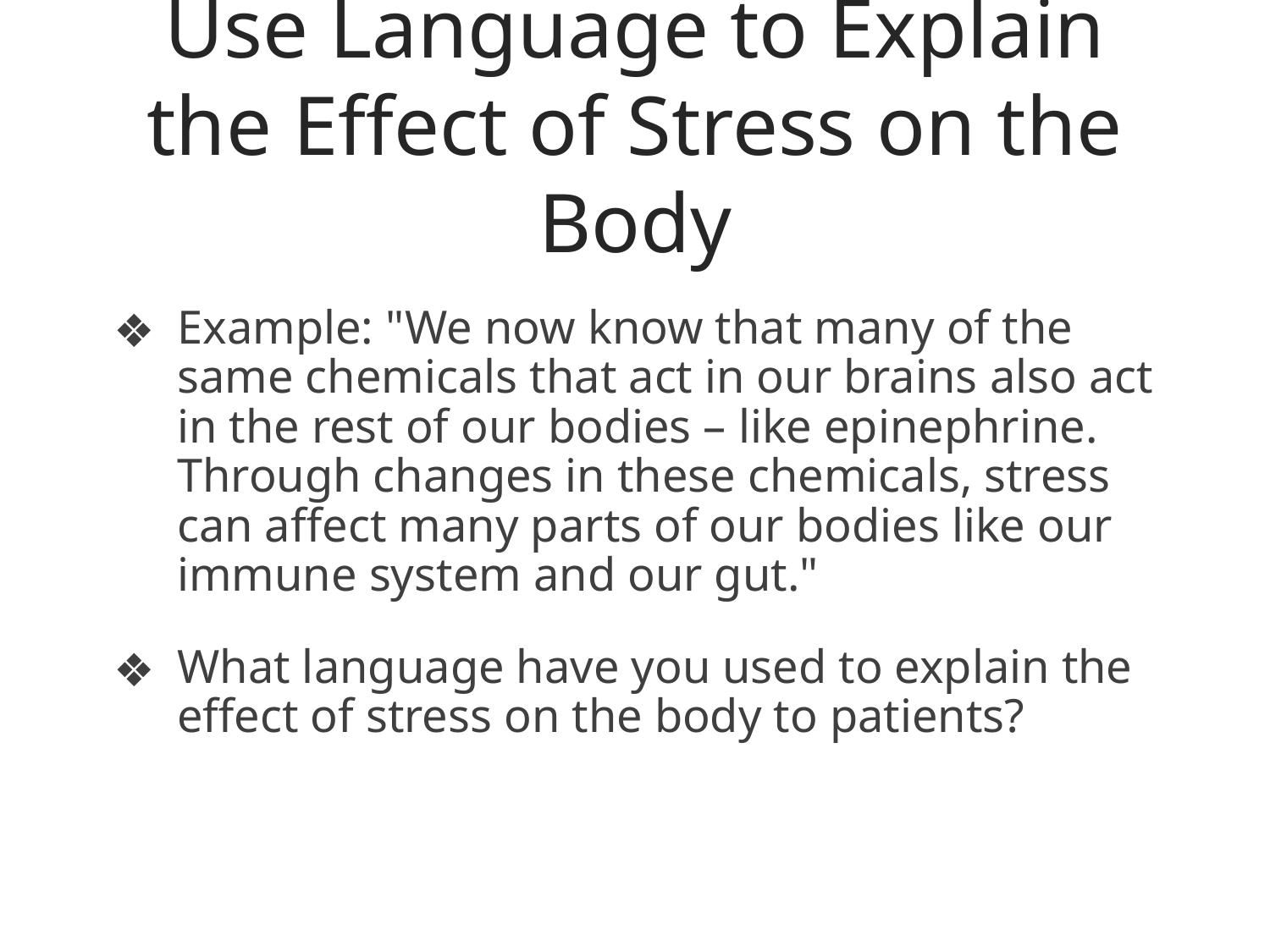

# Use Language to Explain the Effect of Stress on the Body
Example: "We now know that many of the same chemicals that act in our brains also act in the rest of our bodies – like epinephrine. Through changes in these chemicals, stress can affect many parts of our bodies like our immune system and our gut."
What language have you used to explain the effect of stress on the body to patients?

## Slide 7
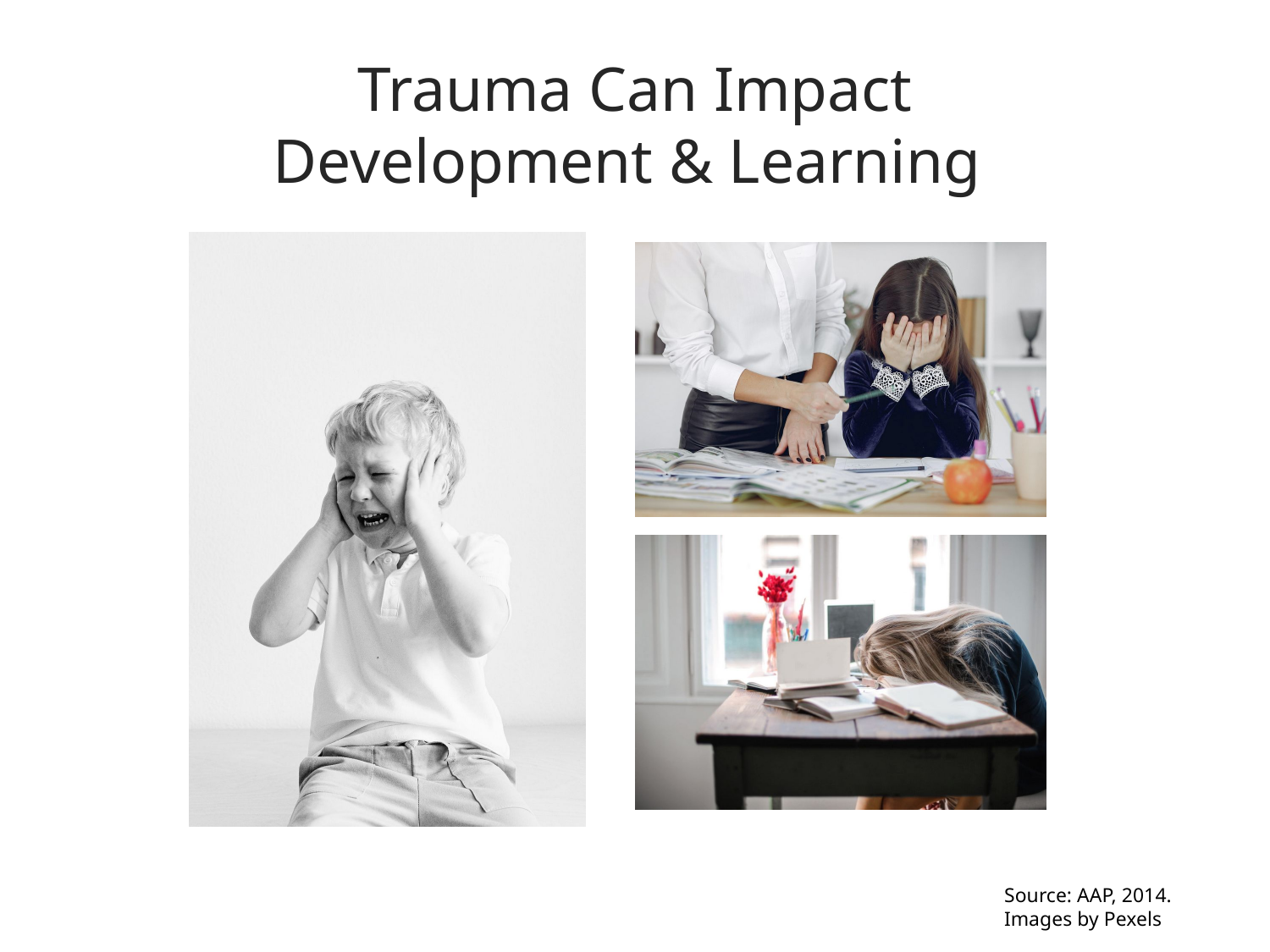

# Trauma Can Impact Development & Learning
Source: AAP, 2014.
Images by Pexels

## Slide 8
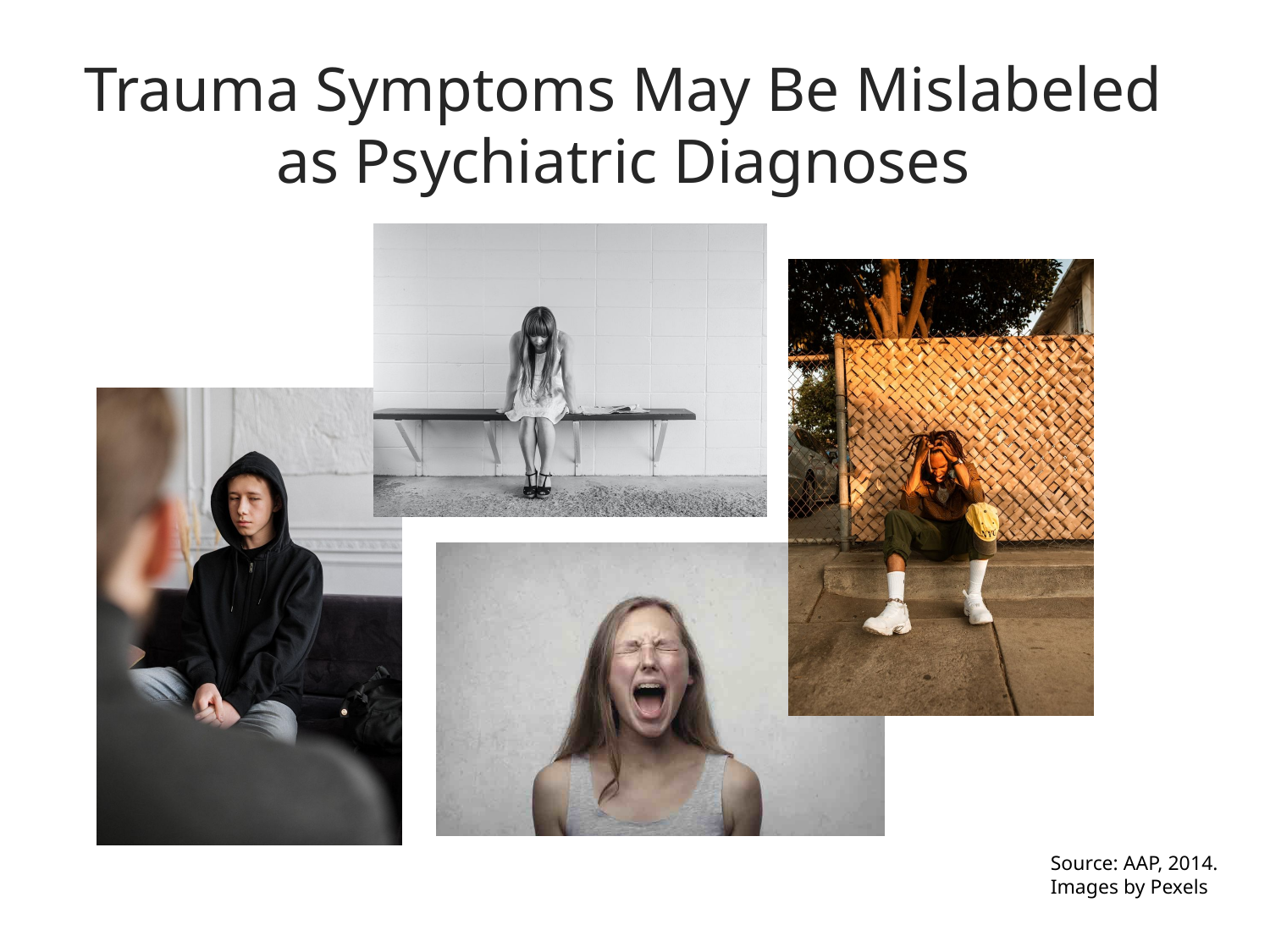

# Trauma Symptoms May Be Mislabeled as Psychiatric Diagnoses
Source: AAP, 2014.
Images by Pexels

## Slide 9
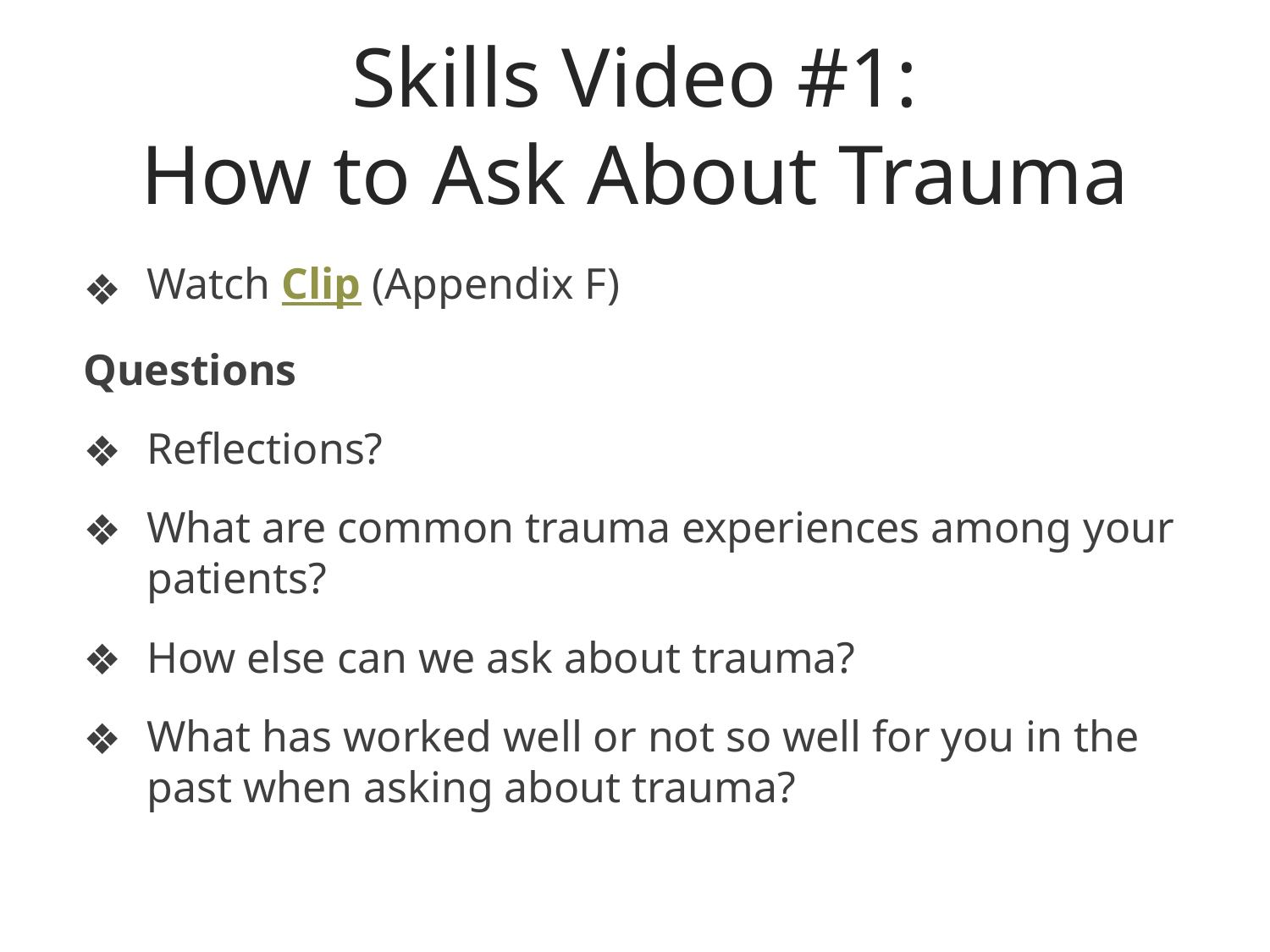

# Skills Video #1: How to Ask About Trauma
Watch Clip (Appendix F)
Questions
Reflections?
What are common trauma experiences among your patients?
How else can we ask about trauma?
What has worked well or not so well for you in the past when asking about trauma?

## Slide 10
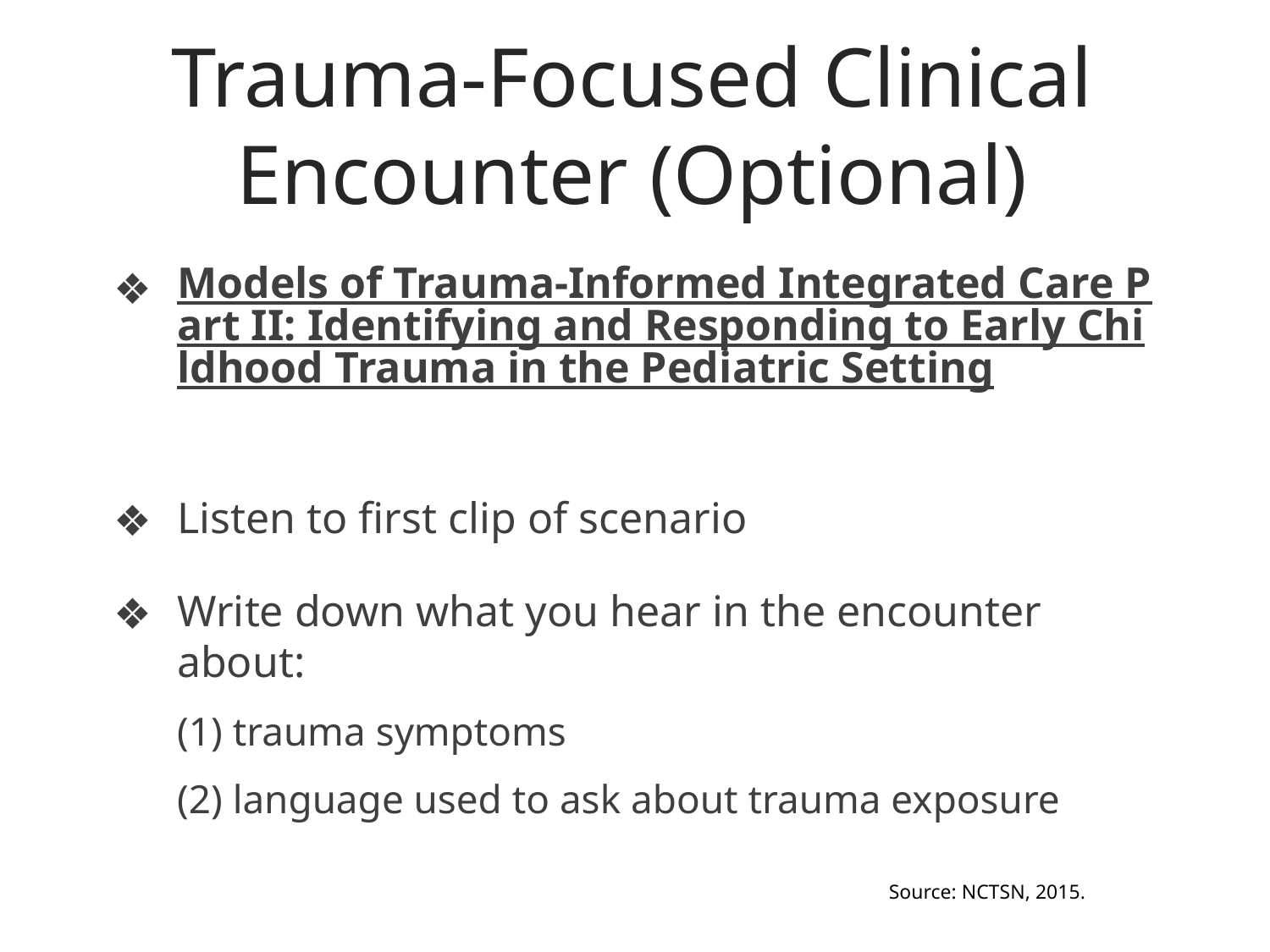

# Trauma-Focused Clinical Encounter (Optional)
Models of Trauma-Informed Integrated Care Part II: Identifying and Responding to Early Childhood Trauma in the Pediatric Setting
Listen to first clip of scenario
Write down what you hear in the encounter about:
(1) trauma symptoms
(2) language used to ask about trauma exposure
Source: NCTSN, 2015.

## Slide 11
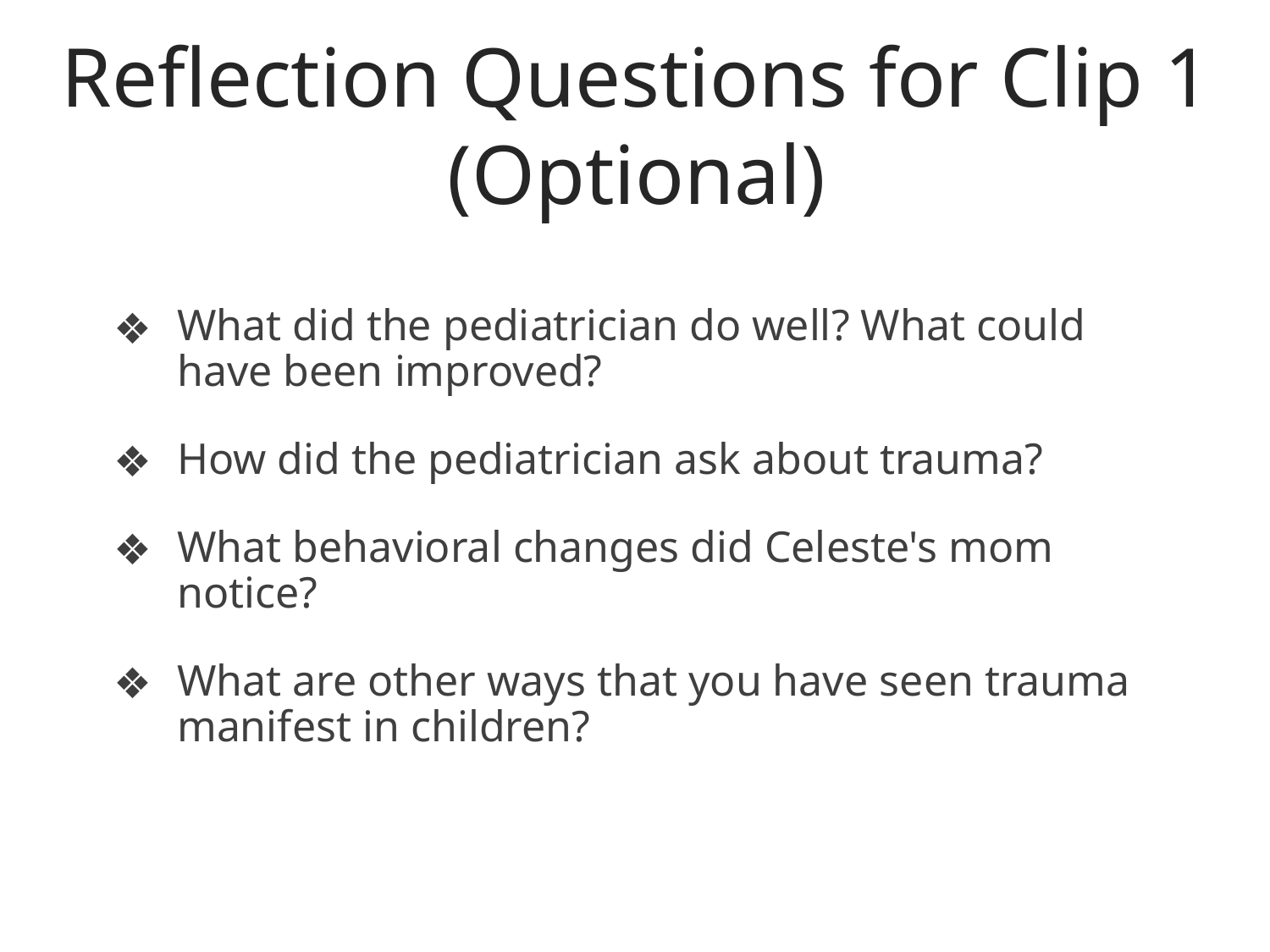

# Reflection Questions for Clip 1 (Optional)
What did the pediatrician do well? What could have been improved?
How did the pediatrician ask about trauma?
What behavioral changes did Celeste's mom notice?
What are other ways that you have seen trauma manifest in children?

## Slide 12
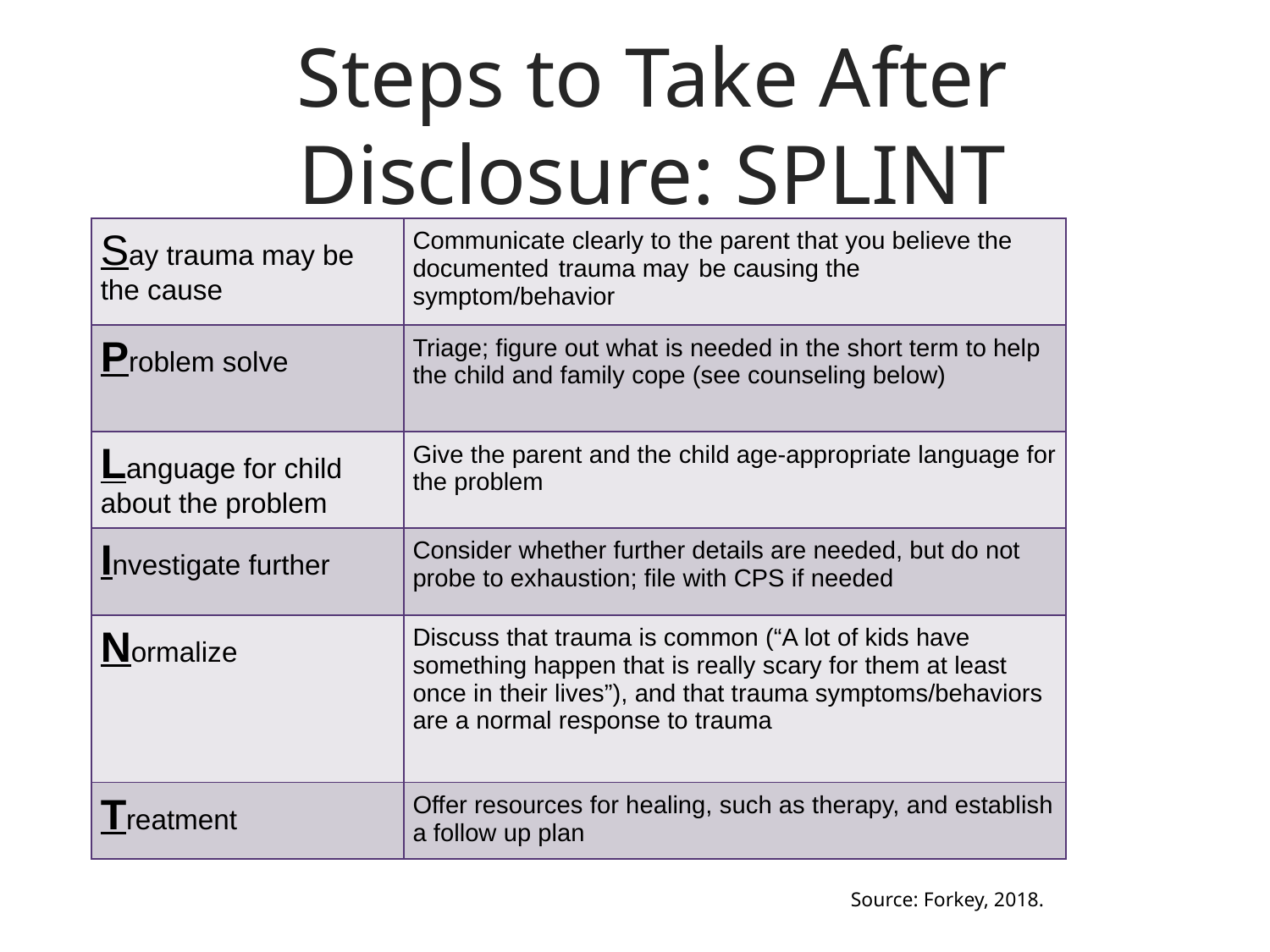

# Steps to Take After Disclosure: SPLINT
| Say trauma may be the cause | Communicate clearly to the parent that you believe the documented  trauma may  be causing the symptom/behavior |
| --- | --- |
| Problem solve | Triage; figure out what is needed in the short term to help the child and family cope (see counseling below) |
| Language for child about the problem | Give the parent and the child age-appropriate language for the problem |
| Investigate further | Consider whether further details are needed, but do not probe to exhaustion; file with CPS if needed |
| Normalize | Discuss that trauma is common (“A lot of kids have something happen that is really scary for them at least once in their lives”), and that trauma symptoms/behaviors are a normal response to trauma |
| Treatment | Offer resources for healing, such as therapy, and establish a follow up plan |
Source: Forkey, 2018.

## Slide 13
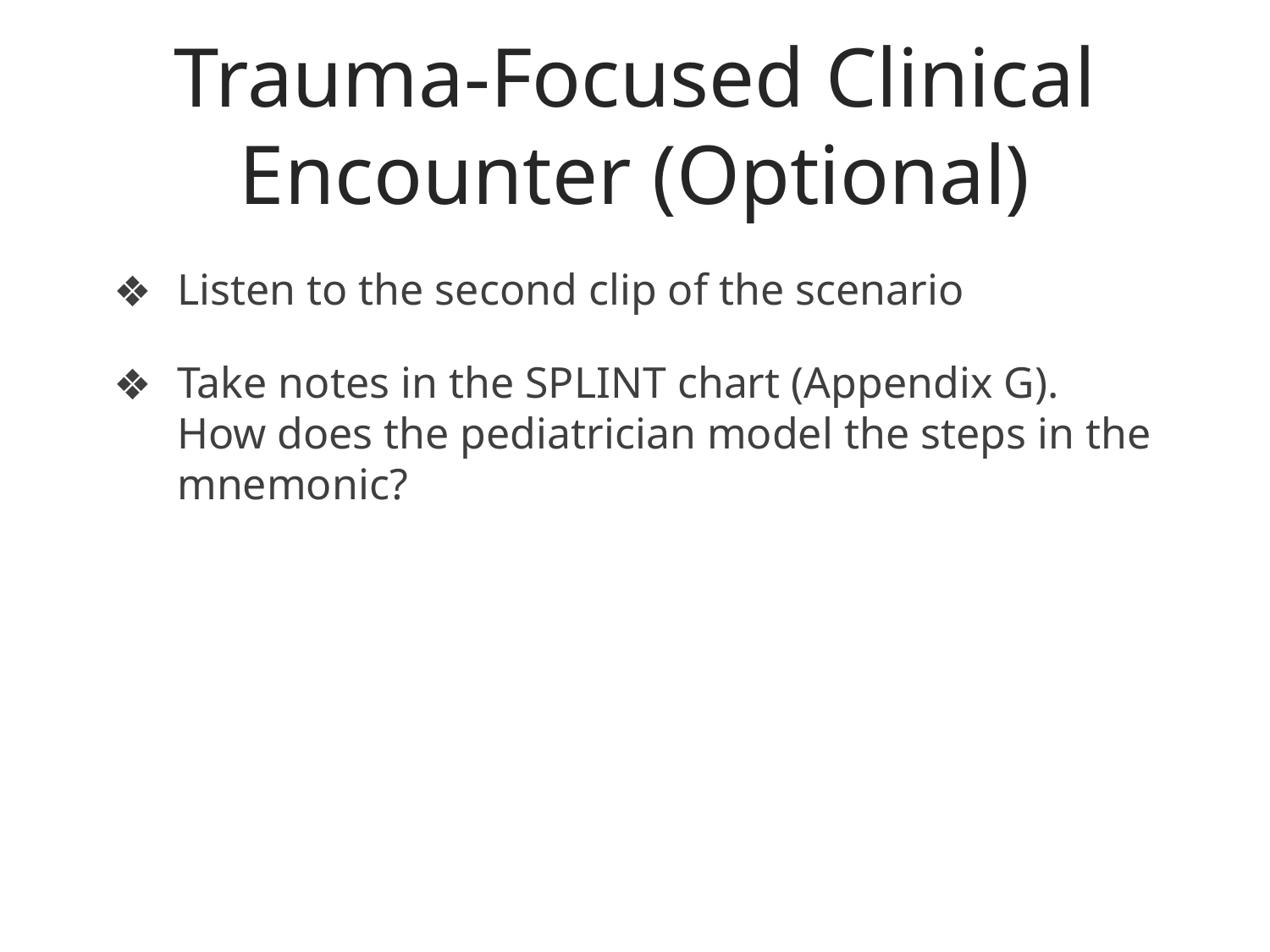

# Trauma-Focused Clinical Encounter (Optional)
Listen to the second clip of the scenario
Take notes in the SPLINT chart (Appendix G). How does the pediatrician model the steps in the mnemonic?

## Slide 14
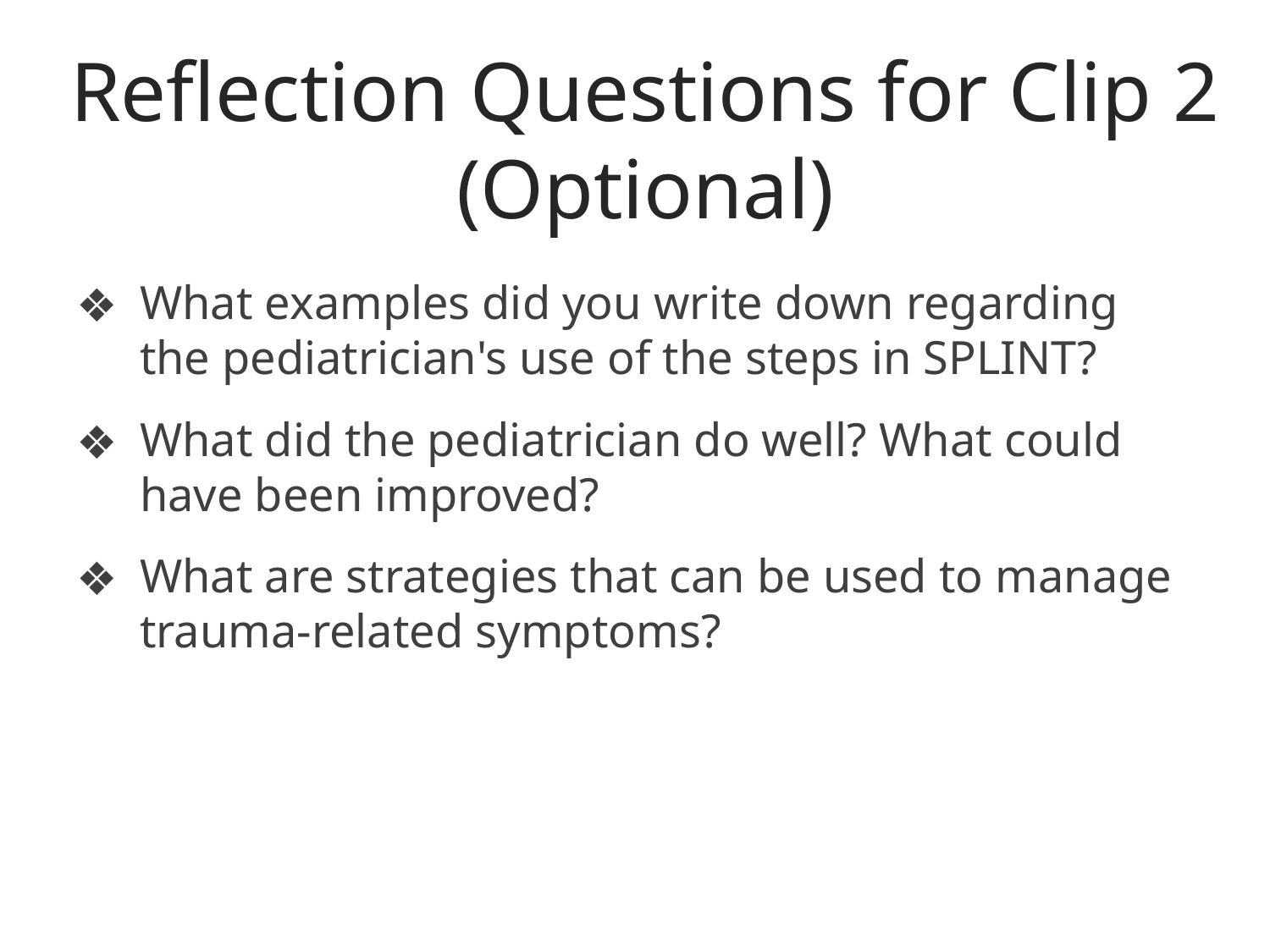

# Reflection Questions for Clip 2 (Optional)
What examples did you write down regarding the pediatrician's use of the steps in SPLINT?
What did the pediatrician do well? What could have been improved?
What are strategies that can be used to manage trauma-related symptoms?

## Slide 15
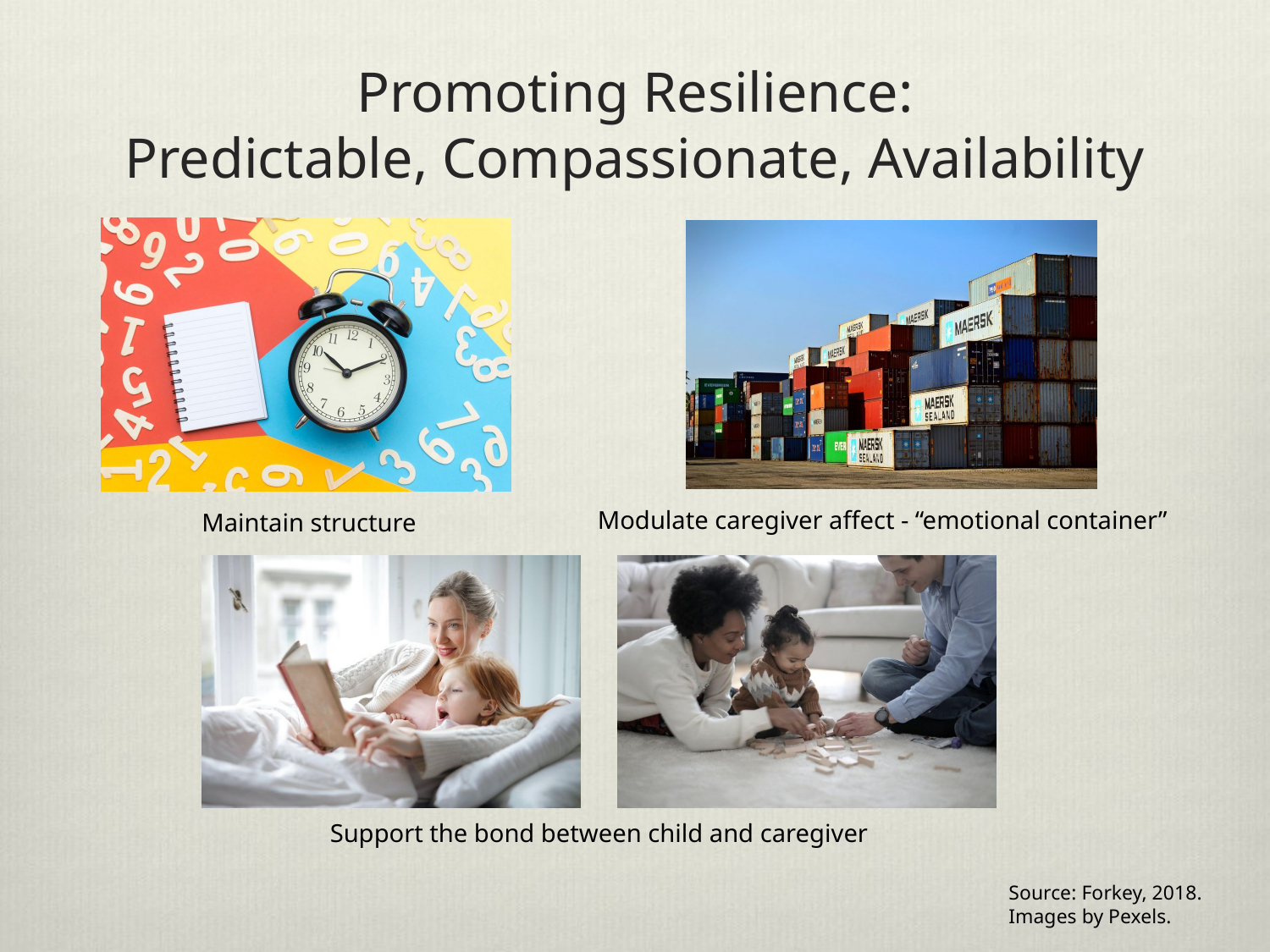

# Promoting Resilience:
Predictable, Compassionate, Availability
Modulate caregiver affect - “emotional container”
Maintain structure
Support the bond between child and caregiver
Source: Forkey, 2018.
Images by Pexels.

## Slide 16
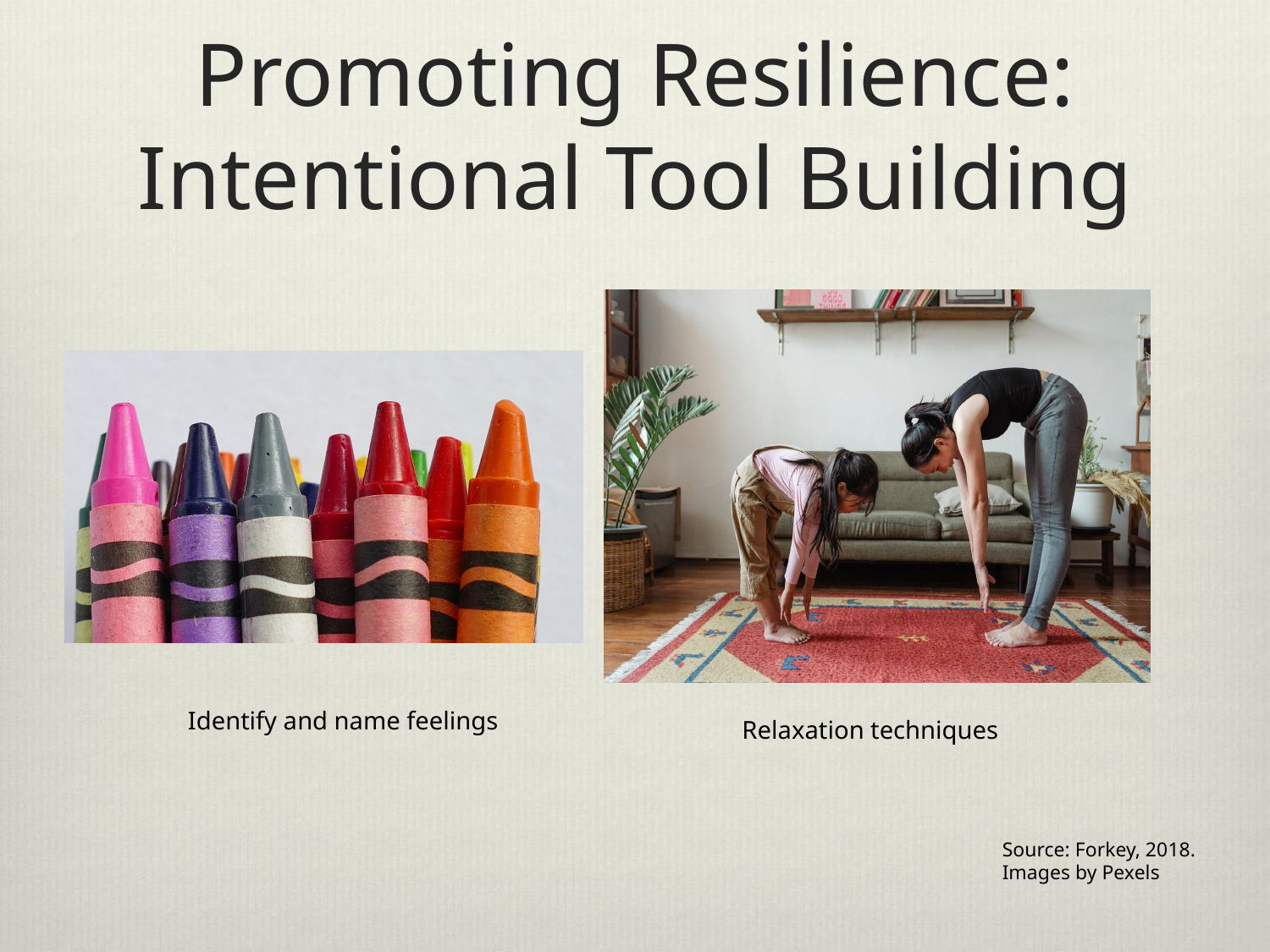

# Promoting Resilience: Intentional Tool Building
Identify and name feelings
Relaxation techniques
Source: Forkey, 2018.
Images by Pexels

## Slide 17
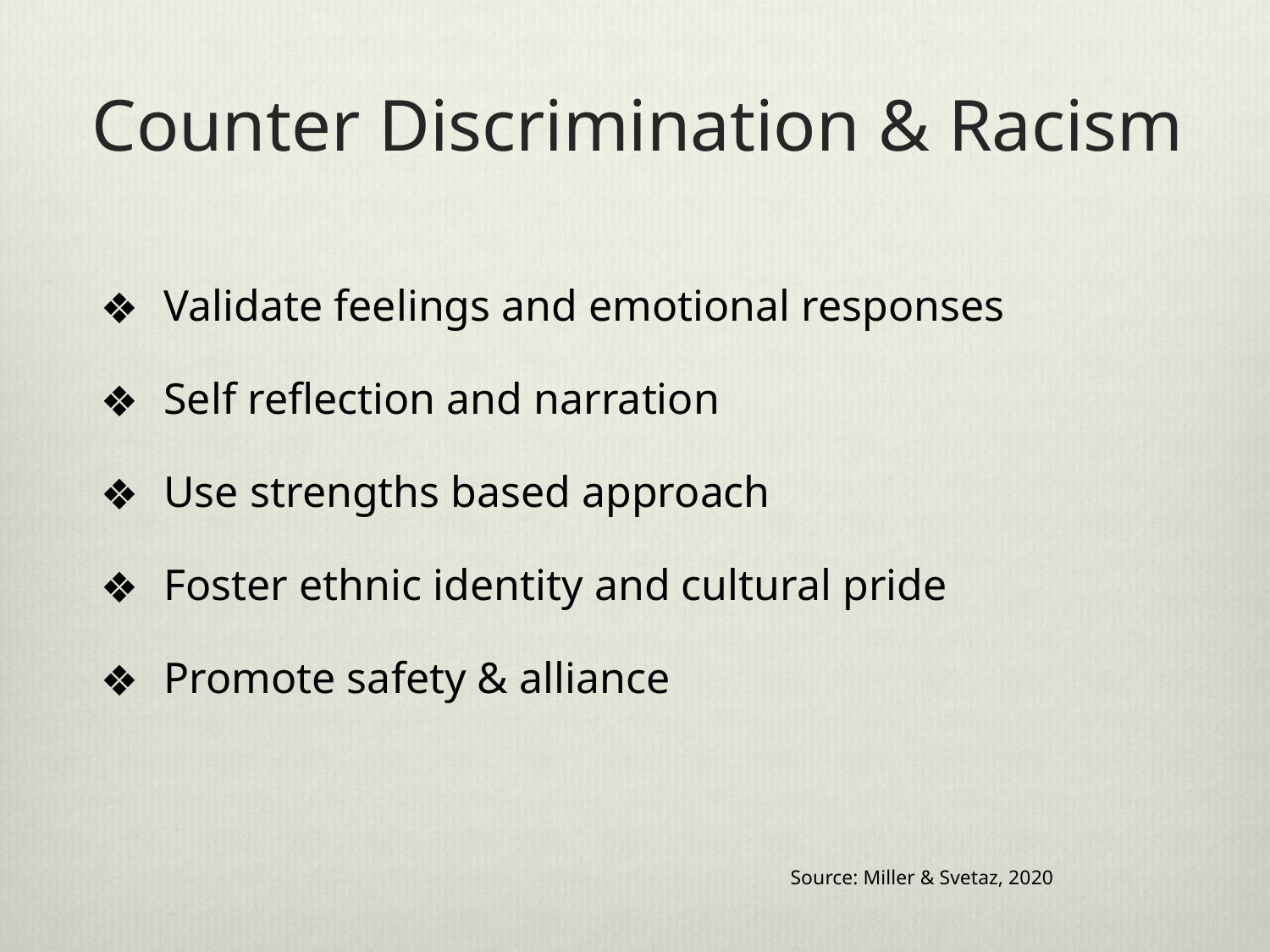

# Counter Discrimination & Racism
Validate feelings and emotional responses
Self reflection and narration
Use strengths based approach
Foster ethnic identity and cultural pride
Promote safety & alliance
Source: Miller & Svetaz, 2020

## Slide 18
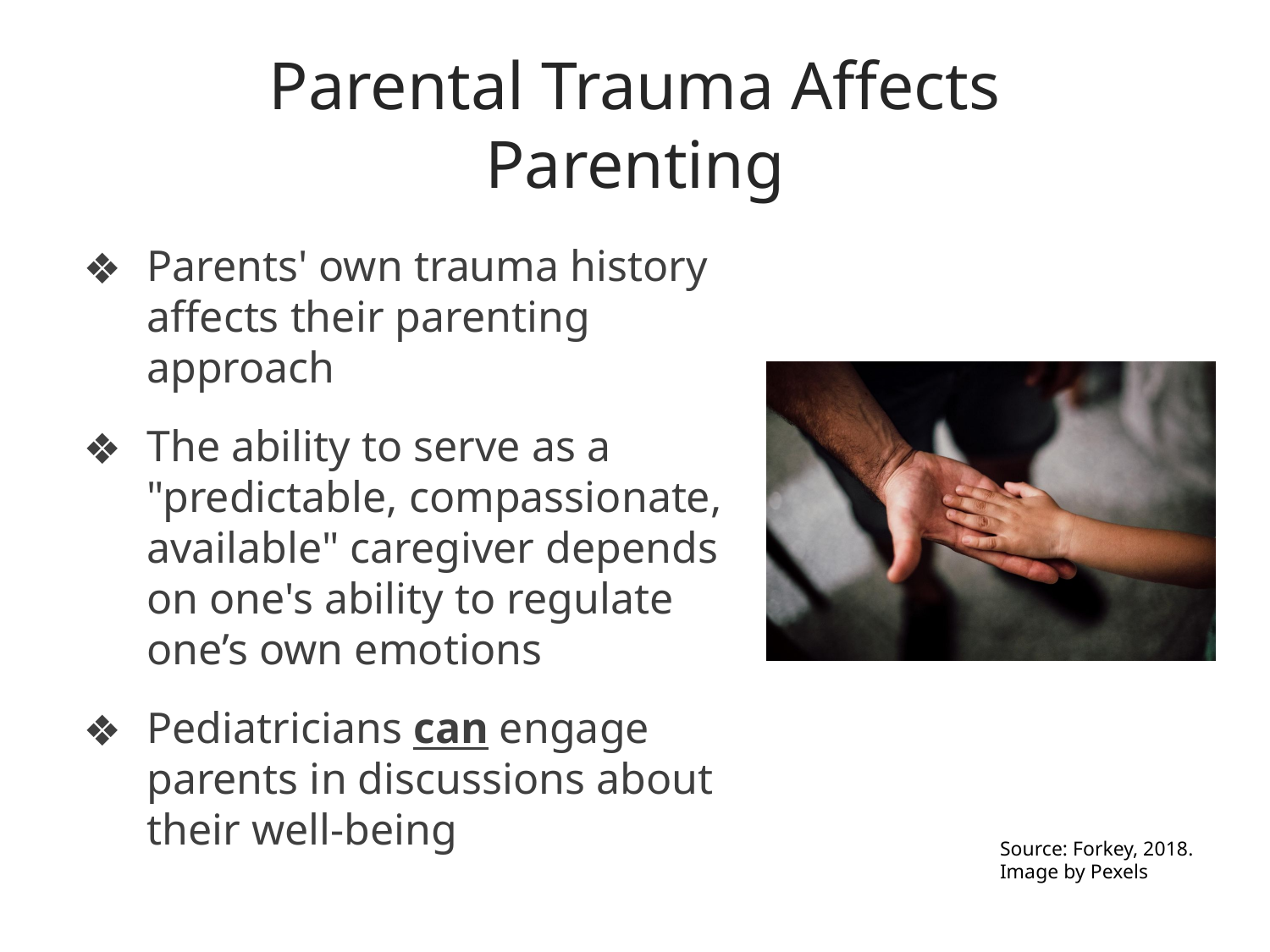

# Parental Trauma Affects Parenting
Parents' own trauma history affects their parenting approach
The ability to serve as a "predictable, compassionate, available" caregiver depends on one's ability to regulate one’s own emotions
Pediatricians can engage parents in discussions about their well-being
Source: Forkey, 2018.
Image by Pexels

## Slide 19
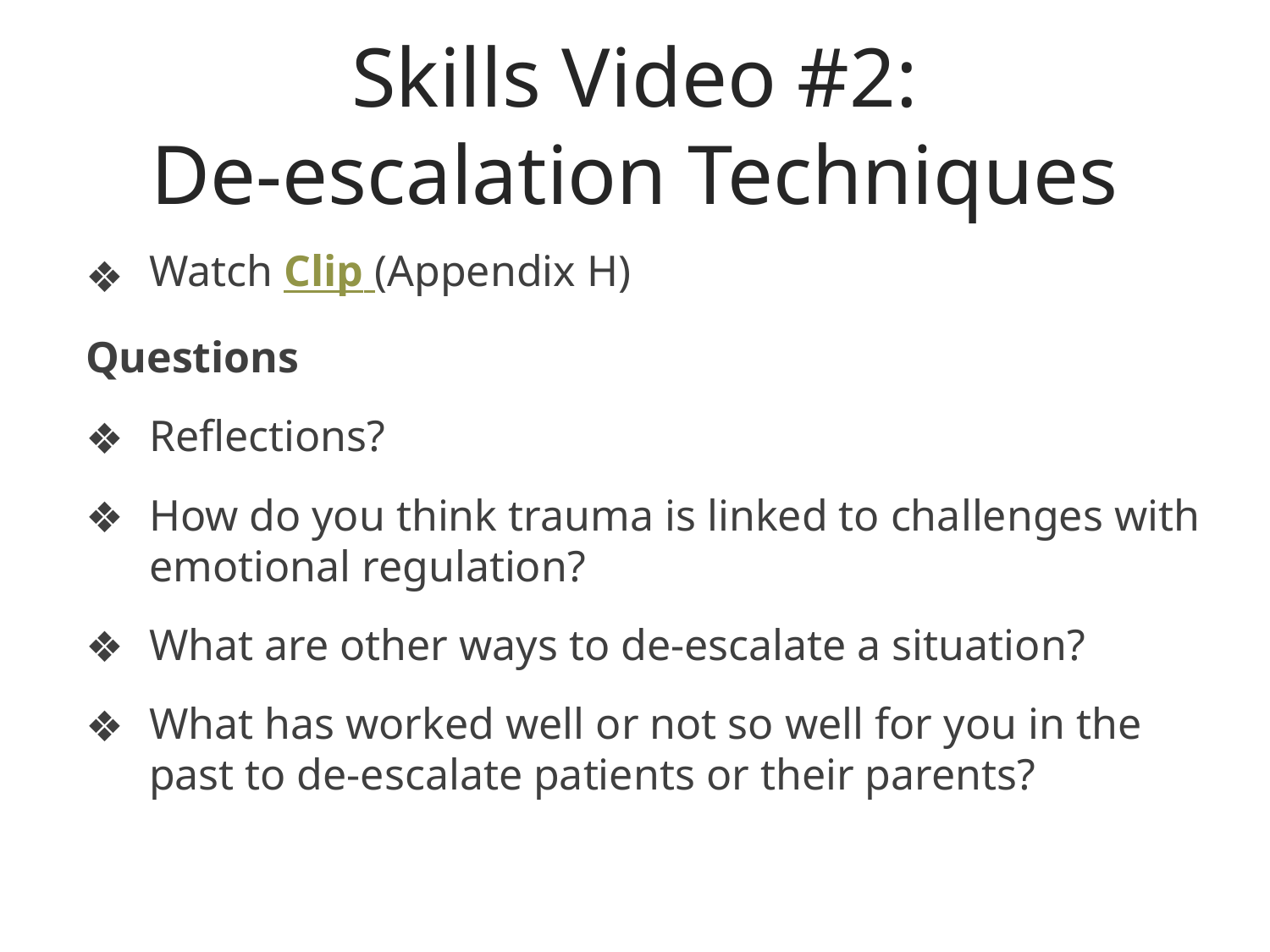

# Skills Video #2: De-escalation Techniques
Watch Clip (Appendix H)
Questions
Reflections?
How do you think trauma is linked to challenges with emotional regulation?
What are other ways to de-escalate a situation?
What has worked well or not so well for you in the past to de-escalate patients or their parents?

## Slide 20
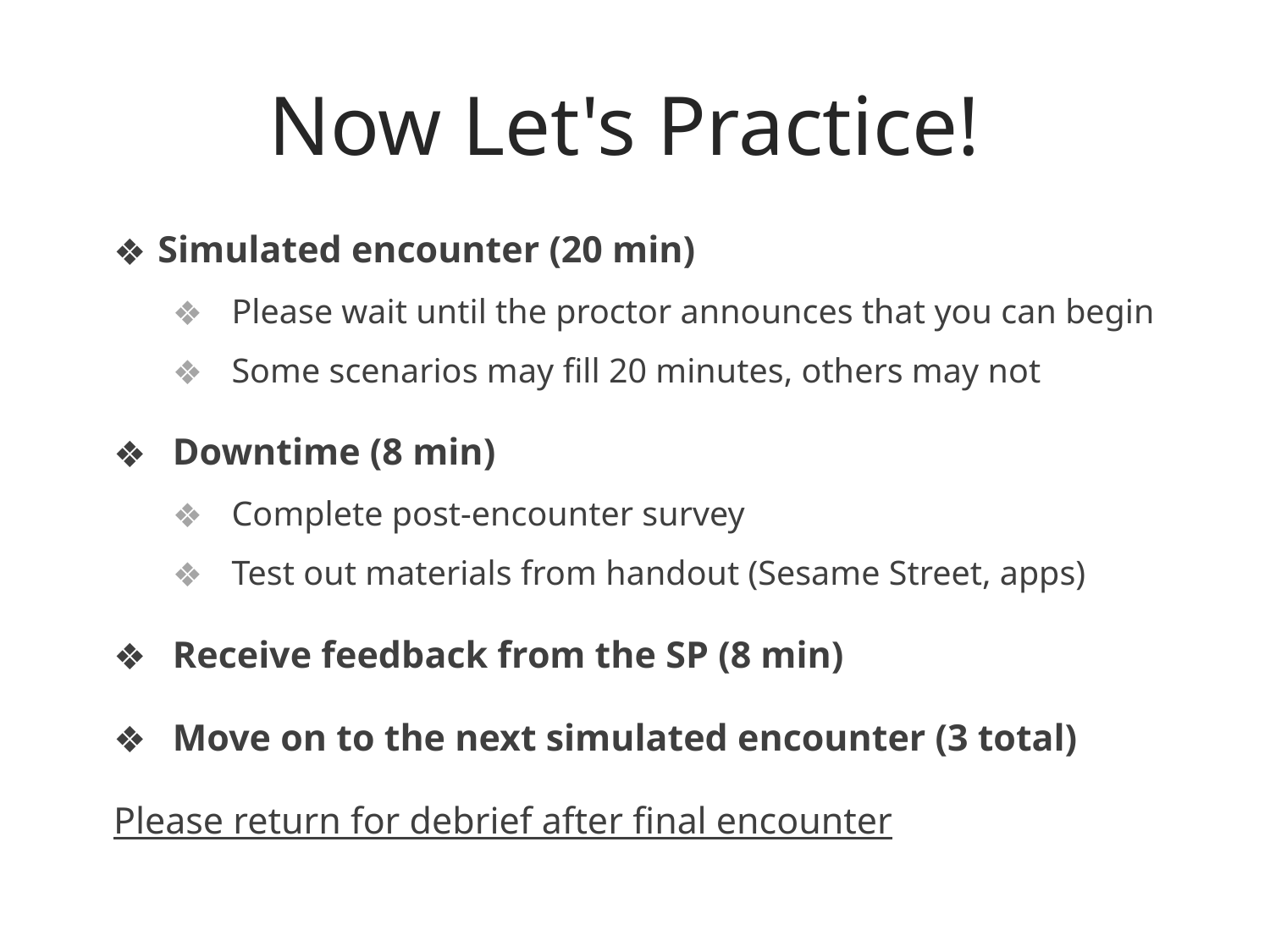

# Now Let's Practice!
Simulated encounter (20 min)
Please wait until the proctor announces that you can begin
Some scenarios may fill 20 minutes, others may not
Downtime (8 min)
Complete post-encounter survey
Test out materials from handout (Sesame Street, apps)
Receive feedback from the SP (8 min)
Move on to the next simulated encounter (3 total)
Please return for debrief after final encounter

## Slide 21
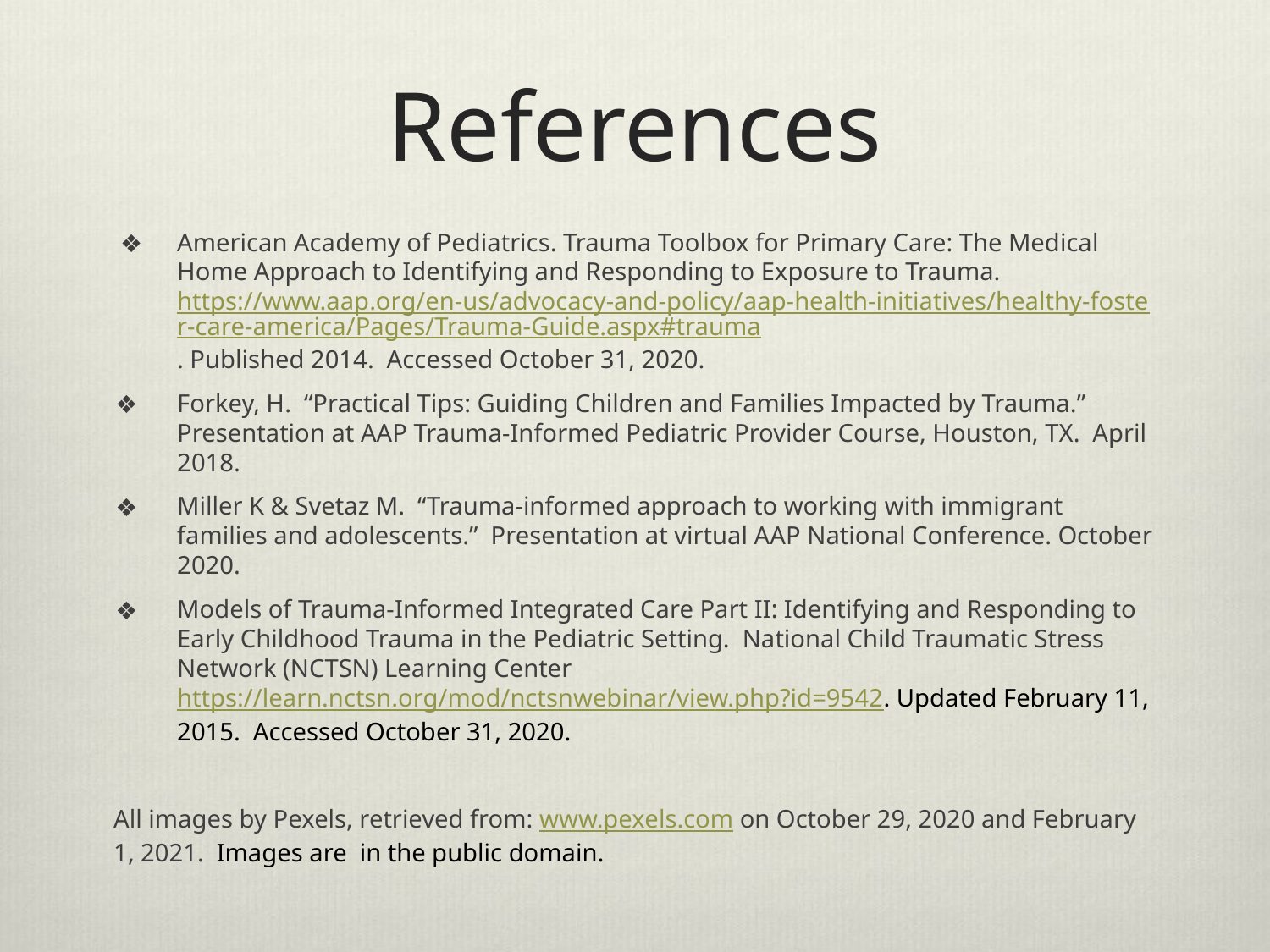

# References
American Academy of Pediatrics. Trauma Toolbox for Primary Care: The Medical Home Approach to Identifying and Responding to Exposure to Trauma. https://www.aap.org/en-us/advocacy-and-policy/aap-health-initiatives/healthy-foster-care-america/Pages/Trauma-Guide.aspx#trauma. Published 2014. Accessed October 31, 2020.
Forkey, H. “Practical Tips: Guiding Children and Families Impacted by Trauma.” Presentation at AAP Trauma-Informed Pediatric Provider Course, Houston, TX. April 2018.
Miller K & Svetaz M. “Trauma-informed approach to working with immigrant families and adolescents.” Presentation at virtual AAP National Conference. October 2020.
Models of Trauma-Informed Integrated Care Part II: Identifying and Responding to Early Childhood Trauma in the Pediatric Setting. National Child Traumatic Stress Network (NCTSN) Learning Center https://learn.nctsn.org/mod/nctsnwebinar/view.php?id=9542. Updated February 11, 2015. Accessed October 31, 2020.
All images by Pexels, retrieved from: www.pexels.com on October 29, 2020 and February 1, 2021. Images are in the public domain.
