# Supplementary material for: Addressing Trauma and Building Resilience in Children and Families: Standardized Patient Cases for Pediatric Residents
Source: MedEdPORTAL. 2021 Nov 8;17:11193. doi: 10.15766/mep_2374-8265.11193 (PMC8592119; doi:10.15766/mep_2374-8265.11193)
Supplement: Supplementary file 1 — Case 1.docxCase 2.docxCase 3.docxResource Packet.docxOrientation Slides.pptxWays to Ask About Trauma.mp4NCTSN Encounter Learner Handout.docxDe-escalation Strategies.mp4Scenario 1 Evaluation Checklist.docxScenario 2 Evaluation Checklist.docxScenario 3 Evaluation Checklist.docxDebrief Instructions.docxPresurvey.docxPostsurvey.docxEncounter-Specific Survey.docx [file mep_2374-8265.11193-s001.zip › G. NCTSN Encounter Learner Handout.docx]

NCTSN Trauma-Focused Clinical Encounter Part #1 – Trauma Symptoms & Asking about Trauma

1) What are the symptoms that Celeste’s mother brings up in her conversation with the pediatrician?

2) What language does the pediatrician use to explore whether trauma might be the cause of these symptoms?

NCTSN Trauma-Focused Clinical Encounter Part #2 – Steps to Take After Disclosure of Trauma: SPLINT

| **Steps** | **Explanation** | **How does the pediatrician take these steps in the clinical encounter?** |
| --- | --- | --- |
| **Say trauma may be the cause** | Communicate clearly to the parent that you believe the documented trauma may be causing the symptom/behavior |  |
| **Problem solve** | Triage; figure out what is needed in the short term to help the child and family cope (see counseling below) |  |
| **Language for child about the problem** | Give the parent and the child age-appropriate language for the problem |  |
| **Investigate further** | Consider whether further details are needed, but do not probe to exhaustion; file with CPS if needed |  |
| **Normalize** | Discuss that trauma is common and that trauma symptoms/behaviors are a normal response to trauma |  |
| **Treatment** | Offer resources for healing, such as therapy, and establish a follow up plan |  |

Author adapted from: Forkey H. Practical tips: Guiding children and families affected by trauma. Oral presentation at: American Academy of Pediatrics Trauma-Informed Pediatric Provider Course; April, 2018; Houston, TX.
