# Supplementary material for: Addressing Trauma and Building Resilience in Children and Families: Standardized Patient Cases for Pediatric Residents
Source: MedEdPORTAL. 2021 Nov 8;17:11193. doi: 10.15766/mep_2374-8265.11193 (PMC8592119; doi:10.15766/mep_2374-8265.11193)
Supplement: Supplementary file 1 — Case 1.docxCase 2.docxCase 3.docxResource Packet.docxOrientation Slides.pptxWays to Ask About Trauma.mp4NCTSN Encounter Learner Handout.docxDe-escalation Strategies.mp4Scenario 1 Evaluation Checklist.docxScenario 2 Evaluation Checklist.docxScenario 3 Evaluation Checklist.docxDebrief Instructions.docxPresurvey.docxPostsurvey.docxEncounter-Specific Survey.docx [file mep_2374-8265.11193-s001.zip › J. Scenario 2 Evaluation Checklist.docx]

**Interpersonal and Communication Skills Checklist for Scenario #2**

| 1. **Opening the Interview – establishing trust and rapport**  - Resident asks an open-ended question to elicit more information after you note that things are going on in the family - You feel comfortable expressing your concerns about why you haven’t discussed this issue with Danny | | | | |
| --- | --- | --- | --- | --- |
| **1**  *I did not want to trust the resident with my personal history* | **2** | **3**  *I felt somewhat comfortable, but was hesitant to fully express my personal history* | **4** | **5**  *I felt a strong culture of trust and rapport with the resident* |
| 1. **Addressing major parent concerns**  - You are able to explain that your main concern is whether or not you should tell Danny - Questions are open-ended and give you the opportunity to expand more about the issues related to communicating with Danny | | | | |
| **1**  *I was not able to address my main concern; the resident immediately started talking about ACE* | **2** | **3**  *My main concern was partially addressed* | **4** | **5**  *I was able to explain most/all of my main concern, and the resident addressed this concern and then made the connection to ACE* |
| 1. **Connection of adverse childhood experiences and health**  - Explains how trauma experiences can impact health - Relates this connection to Danny’s behavior and somatic symptoms | | | | |
| **1**  *The connection wasn’t explained to me at all* | **2** | **3**  *The connection was partially explained in broad terms, but didn’t apply to me specifically* | **4** | **5**  *The connection was fully explained, and I understood the reasons for the questions* |
| 1. **Providing resources to families**  - Resources were provided to practice emotion sharing and communication - Clear explanation of how to utilize the resources provided | | | | |
| **1**  *Resources were not discussed* | **2** | **3**  *Some resources, but did not fully explain how to utilize these resources* | **4** | **5**  *Multiple resources provided. Explained how to utilize these to help Danny* |
| 1. **Establish plan for how to employ resilience strategies**  - Clear explanation of how parent-child relationship and emotional intelligence can build resilience - I felt included in establishing strategies I can use in my everyday life with Danny - The resident collaborated with me in creating a plan | | | | |
| **1**  *The resident listed strategies, rather than having a discussion with me* | **2** | **3**  *I felt partially included in a discussion* | **4** | **5**  *I was able to fully participate in a collaboration discussion about resilience factors in my child* |
| 1. **Plan for follow up and next steps**  - Established specific goals and next steps - Clarified plan for follow up | | | | |
| **1**  *I was able to understand little or none of what would happen next* | **2** | **3**  *I was able to understand some of what would happen next* | **4** | **5**  *I was able to understand most or all of what would happen next* |
| 1. **Supporting My Emotions**  - Asked me to talk more about a stated emotion - Recognized and asked me about an emotion implied through tone of voice, facial expression or other body language | | | | |
| **1**  *The learner rarely or never recognized, asked about, or validated my emotions* | **2** | **3**  *The learner sometimes recognized, asked about, or validated my emotions* | **4** | **5**  *The learner almost always or always recognized, asked about, or validated my emotions* |
| 1. **Showing Interest in Me as a Person**  - Showed interest in me as a person when greeting me - Used words that show care and concern for me and Danny throughout the interview - Used tone, pace, eye contact, and posture that show care and concern | | | | |
| **1**  *The learner showed little/no interest or concern in me as a person* | **2** | **3**  *The learner showed some interest or concern in me as a person* | **4** | **5**  *The learner showed strong/very strong interest or concern in me as a person* |
| 1. **Overall Encounter Rating**   How likely would you be to return to this person as your future care giver? | | | | |
| **1**  *I would be not at all likely to return to this person as my future caregiver* | **2** | **3**  *I would be somewhat likely to return to this person as my future caregiver* | **4** | **5**  *I would be very likely to return to this person as my future caregiver* |
