# Supplementary material for: Addressing Trauma and Building Resilience in Children and Families: Standardized Patient Cases for Pediatric Residents
Source: MedEdPORTAL. 2021 Nov 8;17:11193. doi: 10.15766/mep_2374-8265.11193 (PMC8592119; doi:10.15766/mep_2374-8265.11193)
Supplement: Supplementary file 1 — Case 1.docxCase 2.docxCase 3.docxResource Packet.docxOrientation Slides.pptxWays to Ask About Trauma.mp4NCTSN Encounter Learner Handout.docxDe-escalation Strategies.mp4Scenario 1 Evaluation Checklist.docxScenario 2 Evaluation Checklist.docxScenario 3 Evaluation Checklist.docxDebrief Instructions.docxPresurvey.docxPostsurvey.docxEncounter-Specific Survey.docx [file mep_2374-8265.11193-s001.zip › K. Scenario 3 Evaluation Checklist.docx]

**Interpersonal and Communication Skills Checklist for Scenario #3**

| 1. **Opening the Interview – initial de-escalation technique**  - Resident is respectful and keeps voice low and calm - You are given an opportunity to cool off before starting questioning - Resident steps out of the room to give you a safe space and creates a safe space for the interview when he/she returns | | | | |
| --- | --- | --- | --- | --- |
| **1**  *The resident immediately started asking questions or created an unsafe space for the interview* | **2** | **3**  *I felt somewhat comfortable, but the resident still tried to question me without giving me space* | **4** | **5**  *The resident was respectful, and stepped out of the room to give me space. Respected personal space and maintained safety during the interview.* |
| 1. **Introductions**  - The resident clearly introduces themselves and their role - Establishes an environment of trust at the onset of the interview - Sets clear limits | | | | |
| **1**  *The resident did not introduce themselves, and immediately started asking questions. He/she did not set clear limits.* | **2** | **3**  *The resident quickly said their name, and then started asking questions* | **4** | **5**  *The resident calmly introduced themselves, and addressed you in a way that fosters trust . He/she set clear limits (e.g. I can tell how upset you are, but to be able to communicate with you I am going to ask that you not raise your voice or use inappropriate language)* |
| 1. **De-escalation during the interview**  - Resident uses empathetic statements to build rapport - Does not lecture or become threatening when you are upset - Uses active listening, empathetic open-ended or reflective questions to better understand your emotions and experiences - Identifies wants and feelings | | | | |
| **1**  *The resident lectured and/or threatened me. He/she used did not use active listening techniques or identify wants & feelings.* | **2** | **3**  *The resident attempted to understand, but still came across as lecturing.* | **4** | **5**  *The resident used active & open listening (e.g. Tell me if I have this right..) & uses language like, “I see that you are upset. I am glad you are able to share how you are feeling.”* |
| 1. **Body language**  - Uses open body language | | | | |
| **1**  *Used closed or confrontational body language (arms folded, standing face to face, excessive or lack of eye contact)* | **2** | **3**  *Used neutral body language.* | **4** | **5**  *Used open body language (hands visible, arms not folded, standing at an angle, eye contact but not excessive)* |
| 1. **Connection of adverse childhood experiences and health**  - Explains how traumatic experiences with your dad can impact your emotions and actions | | | | |
| **1**  *The connection wasn’t explained to me at all* | **2** | **3**  *The connection was partially explained in broad terms, but didn’t apply to me specifically* | **4** | **5**  *The connection was fully explained, and I understood the reasons for the questions* |
| 1. **Providing resources to adolescent patients**  - Strategies and resources were offered to help with emotional regulation and processing prior trauma - Clear explanation of how to utilize the strategies or resources provided | | | | |
| **1**  *Resources were not discussed* | **2** | **3**  *Some strategies/resources provided, but did not fully explain how to utilize these resources* | **4** | **5**  *Multiple strategies/resources provided. Explained how to utilize these in your life* |
| 1. **Establish plan for how to employ resilience strategies**  - I felt included in establishing a plan for strategies I can use in my everyday life | | | | |
| **1**  *The resident listed strategies, rather than having a discussion with me* | **2** | **3**  *I felt partially included in a discussion* | **4** | **5**  *I was able to fully participate in a collaboration discussion about resilience factors in my life* |
| 1. **Plan for follow up and next steps**  - Established specific goals and next steps - Clarified plan for follow up | | | | |
| **1**  *I was able to understand little or none of what would happen next* | **2** | **3**  *I was able to understand some of what would happen next* | **4** | **5**  *I was able to understand most or all of what would happen next* |
| 1. **Supporting My Emotions**  - Asked me to talk more about a stated emotion - Recognized and asked me about an emotion implied through tone of voice, facial expression or other body language | | | | |
| **1**  *The learner rarely or never recognized, asked about, or validated my emotions* | **2** | **3**  *The learner sometimes recognized, asked about, or validated my emotions* | **4** | **5**  *The learner almost always or always recognized, asked about, or validated my emotions* |
| 1. **Showing Interest in Me as a Person**  - Showed interest in me as a person when greeting me - Used words that show care and concern throughout the interview - Used tone, pace, eye contact, and posture that show care and concern | | | | |
| **1**  *The learner showed little/no interest or concern in me as a person* | **2** | **3**  *The learner showed some interest or concern in me as a person* | **4** | **5**  *The learner showed strong/very strong interest or concern in me as a person* |
| 1. **Overall Encounter Rating**   How likely would you be to return to this person as your future care giver? | | | | |
| **1**  *I would be not at all likely to return to this person as my future caregiver* | **2** | **3**  *I would be somewhat likely to return to this person as my future caregiver* | **4** | **5**  *I would be very likely to return to this person as my future caregiver* |
