# Supplementary material for: Addressing Trauma and Building Resilience in Children and Families: Standardized Patient Cases for Pediatric Residents
Source: MedEdPORTAL. 2021 Nov 8;17:11193. doi: 10.15766/mep_2374-8265.11193 (PMC8592119; doi:10.15766/mep_2374-8265.11193)
Supplement: Supplementary file 1 — Case 1.docxCase 2.docxCase 3.docxResource Packet.docxOrientation Slides.pptxWays to Ask About Trauma.mp4NCTSN Encounter Learner Handout.docxDe-escalation Strategies.mp4Scenario 1 Evaluation Checklist.docxScenario 2 Evaluation Checklist.docxScenario 3 Evaluation Checklist.docxDebrief Instructions.docxPresurvey.docxPostsurvey.docxEncounter-Specific Survey.docx [file mep_2374-8265.11193-s001.zip › L. Debrief Instructions.docx]

**Instructions for Group Debrief Following SP Scenarios**

1. **Arrange chairs in a circle formation**
2. **Explain to participants that the debrief session will give them the opportunity to reflect on the standardized patient scenarios, their experience using strategies and skills discussed in the orientation, and relevance to their clinical work.**
3. **Remind participants that this is a safe space and comments should not be shared outside the group. Acknowledge that it is possible this topic and these encounters may have triggered memories, strong reactions, or a sense of vicarious trauma for participants. Residents should feel comfortable to share as little or as much as they would like. The debrief leader should also have a list of mental health resources for residents to share, if indicated.**
4. **Use the following questions/prompts to guide discussion:**
   1. How did you feel during the scenarios?
   2. What went well during the scenarios?
   3. What challenges came up during the scenarios?
   4. What new tools or strategies did you try out during the scenarios that we discussed earlier today? *(If learners need prompting, remind them of strategies discussed in orientation including: psychoeducation about trauma-related symptoms, language for how to ask about trauma, SPLINT mnemonic, strategies for resilience promotion, de-escalation techniques)*
      1. How did it feel to use this tool or strategy?
      2. What was effective/ineffective about it?
      3. If it went well, how might you incorporate it into your practice?
      4. If it didn’t go well, how might you adapt it or what alternate strategy might you try in the future?
   5. What additional tools or knowledge do you wish you had to address challenges that came up during the scenarios?
   6. What local resources do you know of that can support families experiencing trauma? (*Facilitator may choose to review handout with local resources during discussion)*
   7. What experiences have you had treating patients and families affected by trauma in clinic or on the wards? What resources have worked well for you in the past when addressing these issues?
   8. How does working with trauma-affected patients and families affect you? What do you do to maintain your own resilience while doing this work?
