# Supplementary material for: Addressing Trauma and Building Resilience in Children and Families: Standardized Patient Cases for Pediatric Residents
Source: MedEdPORTAL. 2021 Nov 8;17:11193. doi: 10.15766/mep_2374-8265.11193 (PMC8592119; doi:10.15766/mep_2374-8265.11193)
Supplement: Supplementary file 1 — Case 1.docxCase 2.docxCase 3.docxResource Packet.docxOrientation Slides.pptxWays to Ask About Trauma.mp4NCTSN Encounter Learner Handout.docxDe-escalation Strategies.mp4Scenario 1 Evaluation Checklist.docxScenario 2 Evaluation Checklist.docxScenario 3 Evaluation Checklist.docxDebrief Instructions.docxPresurvey.docxPostsurvey.docxEncounter-Specific Survey.docx [file mep_2374-8265.11193-s001.zip › M. Presurvey.docx]

Trauma & Resilience Sim: Pre-Encounter Survey

Start of Block: Default Question Block

Q1 How often do you consider adverse childhood experiences (ACEs) and/or trauma when evaluating a patient's chief complaint?

- Never (1)
- Rarely (2)
- Sometimes (3)
- Often (4)
- Always (5)

Q1a Please comment on your consideration of ACEs when evaluating a patient's chief complaint.

________________________________________________________________

________________________________________________________________

________________________________________________________________

________________________________________________________________

________________________________________________________________

Q2 How comfortable do you feel discussing ACEs and/or trauma with your patients/parents?

- Very uncomfortable (1)
- Uncomfortable (2)
- Neither comfortable nor uncomfortable (3)
- Comfortable (4)
- Very comfortable (5)

Q3 How comfortable do you feel asking your patients if they have experienced an ACE and/or traumatic event?

- Very uncomfortable (1)
- Uncomfortable (2)
- Neither comfortable nor uncomfortable (3)
- Comfortable (4)
- Very comfortable (5)

Q4 How comfortable do you feel explaining to patients/parents how traumatic experiences impact health?

- Very uncomfortable (1)
- Uncomfortable (2)
- Neither comfortable nor uncomfortable (3)
- Comfortable (4)
- Very comfortable (5)

Q4a Please comment on your comfort discussing traumatic experiences and explaining their impact on health with your patients/parents.

________________________________________________________________

________________________________________________________________

________________________________________________________________

________________________________________________________________

________________________________________________________________

Q5 How comfortable do you feel identifying protective factors and counseling patients/parents on how to foster resilience?

- Very uncomfortable (1)
- Uncomfortable (2)
- Neither comfortable nor uncomfortable (3)
- Comfortable (4)
- Very comfortable (5)

Q5a Please comment on your comfort identifying protective factors and counseling patients/parents on how to foster resilience.

________________________________________________________________

________________________________________________________________

________________________________________________________________

________________________________________________________________

________________________________________________________________

Q6 How comfortable do feel de-escalating an escalated patient or parent?

- Very uncomfortable (1)
- Uncomfortable (2)
- Neither comfortable nor uncomfortable (3)
- Comfortable (4)
- Very comfortable (5)

Q6a Please comment on your comfort de-escalating an escalated patient or parent.

________________________________________________________________

________________________________________________________________

________________________________________________________________

________________________________________________________________

________________________________________________________________

Q7 How would you rate the adequacy of faculty modeling or training that you have received before today's session regarding how to discuss ACEs and/or trauma with patients/parents?

- Poor (1)
- Marginal (2)
- Adequate (3)
- Good (4)
- Superior (5)

Q8 How applicable are communication skills related to ACEs and/or trauma to your work?

- Not at all applicable (1)
- Marginally applicable (2)
- Neutral (3)
- Somewhat applicable (4)
- Very applicable (5)

Q9 What are examples of ACEs and/or trauma experienced by patients in your clinic or on the wards?

________________________________________________________________

________________________________________________________________

________________________________________________________________

________________________________________________________________

________________________________________________________________

Q10 Please share any additional thoughts you have on this topic.

________________________________________________________________

________________________________________________________________

________________________________________________________________

________________________________________________________________

________________________________________________________________

End of Block: Default Question Block
