# Supplementary material for: Addressing Trauma and Building Resilience in Children and Families: Standardized Patient Cases for Pediatric Residents
Source: MedEdPORTAL. 2021 Nov 8;17:11193. doi: 10.15766/mep_2374-8265.11193 (PMC8592119; doi:10.15766/mep_2374-8265.11193)
Supplement: Supplementary file 1 — Case 1.docxCase 2.docxCase 3.docxResource Packet.docxOrientation Slides.pptxWays to Ask About Trauma.mp4NCTSN Encounter Learner Handout.docxDe-escalation Strategies.mp4Scenario 1 Evaluation Checklist.docxScenario 2 Evaluation Checklist.docxScenario 3 Evaluation Checklist.docxDebrief Instructions.docxPresurvey.docxPostsurvey.docxEncounter-Specific Survey.docx [file mep_2374-8265.11193-s001.zip › N. Postsurvey.docx]

Trauma & Resilience Sim: Final Survey

Start of Block: Default Question Block

Q1 After this session, how often do you plan to consider ACEs and/or trauma when evaluating a patient's chief complaint?

- Never (1)
- Rarely (2)
- Sometimes (3)
- Often (4)
- Always (5)

Q1a Please explain.

________________________________________________________________

________________________________________________________________

________________________________________________________________

________________________________________________________________

________________________________________________________________

Q2 After this session, how comfortable do you feel discussing ACEs and/or trauma with your patients/parents?

- Very uncomfortable (1)
- Uncomfortable (2)
- Neither comfortable nor uncomfortable (3)
- Comfortable (4)
- Very comfortable (5)

Q3 After this session, how comfortable do you feel asking your patients if they have experienced an ACE and/or traumatic event?

- Very uncomfortable (1)
- Uncomfortable (2)
- Neither comfortable nor uncomfortable (3)
- Comfortable (4)
- Very comfortable (5)

Q4 After this session, how comfortable do you feel explaining to patients/parents how traumatic experiences impact health?

- Very uncomfortable (1)
- Uncomfortable (2)
- Neither comfortable nor uncomfortable (3)
- Comfortable (4)
- Very comfortable (5)

Q4a Please explain.

________________________________________________________________

________________________________________________________________

________________________________________________________________

________________________________________________________________

________________________________________________________________

Q5 After this session, how comfortable do you feel identifying protective factors and counseling patients/parents on how to foster resilience?

- Very uncomfortable (1)
- Uncomfortable (2)
- Neither comfortable nor uncomfortable (3)
- Comfortable (4)
- Very comfortable (5)

Q5a Please explain.

________________________________________________________________

________________________________________________________________

________________________________________________________________

________________________________________________________________

________________________________________________________________

Q6 After this session, how comfortable do feel de-escalating an escalated patient or parent?

- Very uncomfortable (1)
- Uncomfortable (2)
- Neither comfortable nor uncomfortable (3)
- Comfortable (4)
- Very comfortable (5)

Q6a Please explain.

________________________________________________________________

________________________________________________________________

________________________________________________________________

________________________________________________________________

________________________________________________________________

Q7 How useful do you feel the pre-reading packet was in preparing you for today's session?

- Very useless (1)
- Useless (2)
- Neither useful nor useless (3)
- Useful (4)
- Very useful (5)

Q7a Please explain.  How can we improve the pre-reading packet?

________________________________________________________________

________________________________________________________________

________________________________________________________________

________________________________________________________________

________________________________________________________________

Q24 How useful do you feel the orientation was in preparing you for today's session?

- Very useless (1)
- Useless (2)
- Neither useful nor useless (3)
- Useful (4)
- Very useful (5)

Q25 Please explain.  How can we improve the orientation?

________________________________________________________________

________________________________________________________________

________________________________________________________________

________________________________________________________________

________________________________________________________________

Q26 How useful was the debrief in reviewing what you learned during today's session?

- Very useless (1)
- Useless (2)
- Neither useful nor useless (3)
- Useful (4)
- Very useful (5)

Q27 Please explain.  How can we improve the debrief?

________________________________________________________________

________________________________________________________________

________________________________________________________________

________________________________________________________________

________________________________________________________________

Q9 We welcome any additional feedback about this session.  Please share your thoughts freely below.

________________________________________________________________

________________________________________________________________

________________________________________________________________

________________________________________________________________

________________________________________________________________

End of Block: Default Question Block
